# Community analysis of gut microbiota in hornets, the largest eusocial wasps, *Vespa mandarinia* and *V. simillima*

**DOI:** 10.1038/s41598-019-46388-1

**Published:** 2019-07-08

**Authors:** Shota Suenami, Masaru Konishi Nobu, Ryo Miyazaki

**Affiliations:** 1 Bioproduction Research Institute, The National Institute of Advanced Industrial Science and Technology (AIST), Tsukuba, Japan; 2 Computational Bio Big Data Open Innovation Laboratory (CBBD-OIL), AIST, Tokyo, Japan; 3 Faculty of Life and Environmental Sciences, University of Tsukuba, Tsukuba, Japan

**Keywords:** Microbiome, Symbiosis

## Abstract

Gut microbiota are important for various aspects of host physiology, and its composition is generally influenced by both intrinsic and extrinsic contexts of the host. Social bee gut microbiota composition is simple and highly stable hypothesized to be due to their unique food habit and social interactions. Here, we focused on hornets, the largest of the eusocial wasps – *Vespa mandarinia* and *V. simillima*. Unlike the well-studied honey bees, adult hornets are generally herbivorous but also hunt insects for broods, a unique behavior which could influence their gut microbiota. Analysis of the gut microbiome using 16S rRNA gene sequencing revealed that the two species have simple gut microbiota, composed of seven or eight consistently maintained ‘core’ operational taxonomic units (OTUs). While the two *Vespa* species shared some OTUs, the structures of their gut communities differed. Phylogenetic analysis indicated association of core OTUs with host diet. Intriguingly, prey honey bee gut microbes were detected in the *V. simillima* gut (and to a lesser extent in *V. mandarinia*), suggesting migration of microorganisms from the prey gut. This is the first report uncovering gut microbiome in hornets, giving additional insight into how food habit affects gut microbiota of social insects.

## Introduction

Gut microbiota influences multiple aspects of host, such as food digestion, immune system, behavior, and disease^1^. Development and maintenance of gut microbiota is thus paramount for host health and fitness. Dominant gut microbial populations consistently associated with a given animal species (so-called “core gut microbiota”) likely play an essential role in host physiology via symbiosis, and have been a subject of study across many disciplines for years^2^. In general, gut microbiota composition is influenced by several intrinsic and extrinsic contexts of the host animal, such as diet^3^, physiological state^4^, and genetic background^5,6^. Among these, unique dietary behavior is known to develop specialized gut microbiota. Lower termites, a group of social insects in the order Isoptera, digest wood-derived substrates with the help of symbiotic flagellates in their hindgut^7^. Social insects in the order Hymenoptera possess unique gut microbiota with high stability (e.g., *Cephalotes* ant)^8^ and low diversity (e.g., honey bees, bumble bees, and stingless bees)^9–11^. This is likely dictated by bees and ants’ restricted diet (e.g., herbivorous and pollen-feeding)^8^ and their nature to share food among individuals in a colony. However, the mechanisms that develop such core gut microbiota are not fully understood.

Members of Hymenoptera subfamily Vespinae, eusocial hornets (genus *Vespa*) and wasps (genera *Vespula* and *Dolichovespula*), exhibit behavior distinct from well-studied social insects. Hornets and wasps (collectively called vespines) perform caste-dependent reproduction, but do not have clear age-dependent division of labor in sterile castes: sterile individuals can perform various tasks both inside and outside of their nest in one day^12^. Vespines also have specific food habits. While vespines and honey bees both depend on plant-derived liquids as carbohydrate sources (e.g., tree sap and floral nectar respectively), they obtain amino acids through different strategies – honey bees consume pollen whereas adult vespines hunt various insects (e.g., honey bees and mantises) without consuming, deliver and feed them to larvae, and consume nitrogen-rich saliva that the larva produce in return^13^. Vespines can thus be a good model to further understand how food habit and social interaction influence gut microbiota. Yet, studies of vespine gut microbiota are limited and still preliminary; so far only inter-individual variation in larval gut microbiota of *Vespula germanica*^14^ and microbiota homogeneity among *V. vulgaris* adults^15^ have been investigated. Moreover, both studies employed a low-resolution method for microbiome analysis (i.e., denaturing gradient gel electrophoresis), so the true structure and diversity of the gut microbiota and host-microbiome relationships remain unclear.

**Figure 1:**
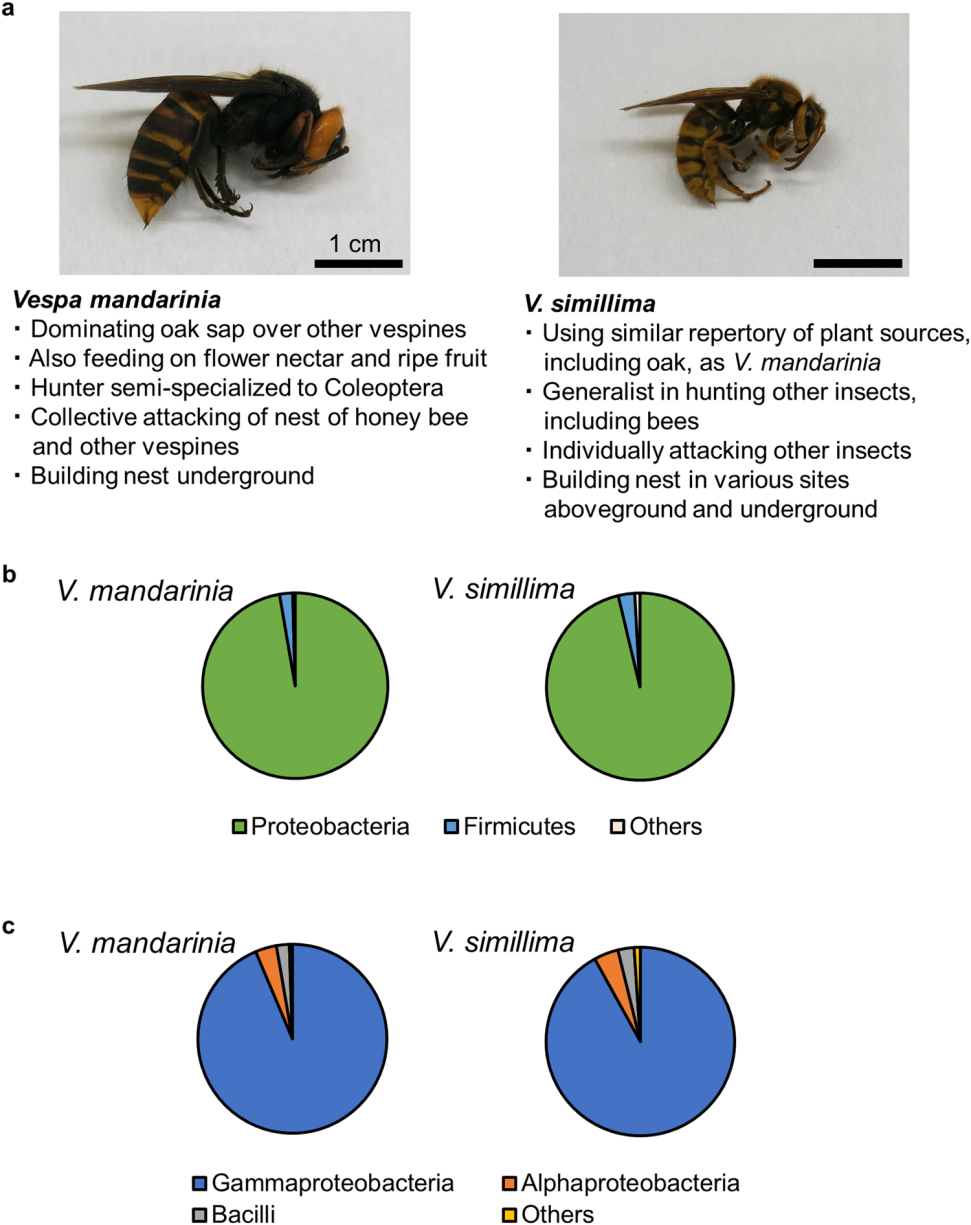
Overview of hornet gut microbiomes. (**a**) Photos of two hornet species analyzed in this study and characteristic ecology of each species are shown. Left, *V. mandarinia*; right, *V. simillima*. (**b**) Averaged relative abundances of OTUs in hornet gut microbiome at the phylum level. (**c**) Same as B but the class level. Taxonomies represented less than 1% are included in ‘Others’. Taxonomic classification was performed based on SILVA. *V. mandarinia*, N = 9; *V. simillima*, N = 8.

In this study, we characterized and compared the gut microbiome in two hornet species, *Vespa mandarinia* (known as Asian giant hornet) and *V. simillima* (known as Japanese yellow hornet) (Fig. 1a), using 16S rRNA gene amplicon sequencing. *V. mandarina* and *V. simillima* inhabit eastern and southern Asia^12,16^. Although the two species follow the general food habit of vespines described above, they have different food preferences. For example, *V. mandarinia* particularly prefers oak sap^12,13^ and tends to hunt Coleoptera^17^, while *V. simillima* is a generalist for insect prey. In addition, *V. mandarinia* collectively attack nests of honey bees and other vespines, including *V. simillima*. We examined whether the gut microbiomes are related to their food habit.

## Results

### Overview of hornet gut microbiota

We collected both *V. mandarinia* and *V. simillima* adults at the National Institute of Advanced Industrial Science and Technology (AIST) and the University of Tokyo (UT) in Japan, and extracted their gut DNAs for 16S rRNA gene sequencing (see Methods). High-throughput sequencing yielded 12627–56357 reads for each sample (Table 1). QIIME 2-based microbial community analysis yielded a total of 465 OTUs. These OTUs represented more than 91% of the observed OTUs for all samples (>95% for all samples except *V. mandarinia* UT-5) based on alpha rarefaction analysis at a sampling depth of 12627 reads (Fig. S1). Thus, subsampling to the minimum sampling depth of this study (12627 reads) was expected to have a minimal effect on the downstream analyses.

**Table 1:** Sequence reads used in this study and rarefaction analysis of the number of OTUs.

Host species	Location	Sample ID	Read count	The number of observed OTUs		
				Total	Average at sampling depth of 12627 reads	Percentage (at depths 12627/Total)
*V. mandarinia*	AIST	1	20543	43	43	100
		2	19115	20	20	100
		3	18307	28	28	100
		4	22719	49	48.5	99
	UT	1	12627	35	35	100
		2	17856	25	24.9	99.6
		3	16431	30	30	100
		4	15399	44	44	100
		5	56357	120	109.4	91.2
*V. simillima*	AIST	1	17092	67	66.9	99.9
		2	21564	42	41.8	99.5
		3	14828	40	40	100
		4	22060	55	54.2	98.5
		5	22981	49	48.7	99.4
	UT	1	34179	183	175.2	95.7
		2	20070	27	26.9	99.6
		3	23006	25	24.9	99.6

Host species, locations of sampling, read counts and rarefaction analysis in terms of the number of observed OTUs are shown. The OTU numbers at sampling depth of 12627 reads are average of ten iterations. AIST, the National Institute of Advanced Industrial Science and Technology; UT, the University of Tokyo.

**Figure 2:**
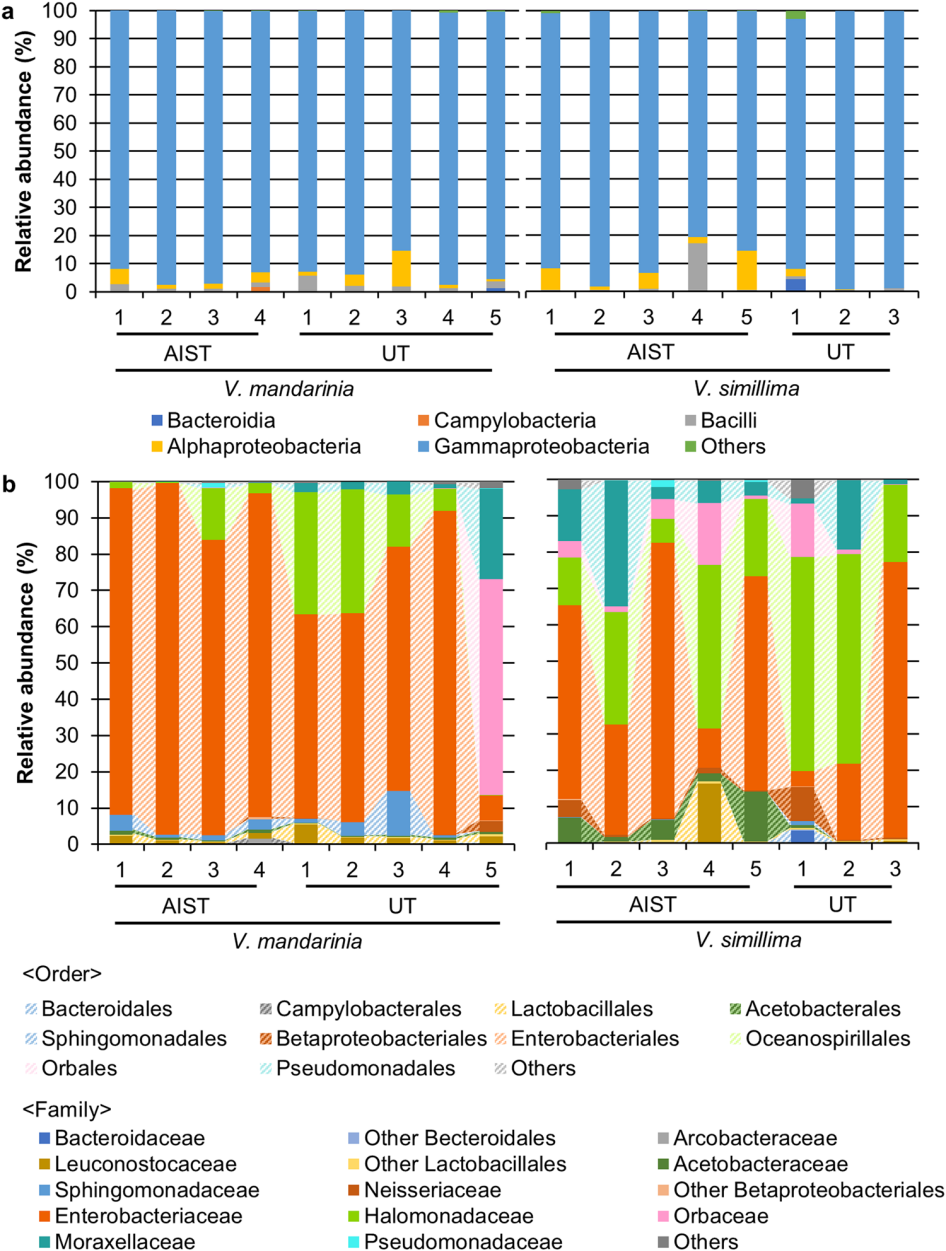
Relative abundance of OTUs in individual gut microbiome. (**a**) Bar plots on relative abundances of OTUs annotated at the class level. (**b**) Same as A but the order and family levels. Order is represented by area chart, while family is shown by bar plots. Taxonomies whose abundance was less than 1% are included in ‘Others’. Taxonomic classification was performed based on SILVA. AIST, the National Institute of Advanced Industrial Science and Technology; UT, the University of Tokyo.

Based on phylogenetic classification using the SILVA 16S rRNA gene database^18,19^, most reads were classified as Proteobacteria (97.3% and 96.3% of OTUs in *V. mandarinia* and *V. simillima*, respectively; Fig. 1b), and Firmicutes was the second most abundant phylum (2.28% and 2.83% in *V. mandarinia* and *V. simillima*, respectively; Fig. 1b). Other phyla comprised less than 1% of the reads for each sample. At the class level, Gammaproteobacteria, Alphaproteobacteria, and Bacilli were the most abundant, comprising 92.0–93.7%, 3.62–4.19%, and 2.21–2.77% of the gut microbiomes in *V. mandarinia* and *V. simillima* respectively (Fig. 1c). While the two hornet species had similar gut microbiomes at the class level (Fig. 2a), their composition clearly differed at the order and family level (Fig. 2b). Among Gammaproteobacteria in *V. mandarinia*, Enterobacteriales (average 70.6 ± 28.1%) and Oceanospirallales (12.0 ± 13.6%) were the two most dominant orders. On the other hand, *V. simillima* had diverse Enterobacteriales abundances (41.3 ± 28.4%) and had higher abundances of other non-Enterobacteriales orders, Oceanospirillales (31.8 ± 19.9%,), Orbales (5.74 ± 6.57%,), and Pseudomonadales (10.8 ± 11.5%). As for Alphaproteobacteria, Sphingomonadales predominated in *V. mandarinia* (3.05 ± 3.78%), while Acetobacterales predominated in *V. simillima* (3.79 ± 4.74%). Family-level community structure was mostly similar to order-level since all dominant orders derived from single families. Pseudomonadales was an exception and consisted of two families, Moraxellaceae and Pseudomonadaceae, former of which is more abundant in *V. simillima* (10.5 ± 11.7% and 0.30 ± 0.66% for Moraxellaceae and Pseudomonadaceae, respectively). Only *V. mandarinia* UT-5 showed very different composition from other conspecifics. Although natural variation among individuals is a possible explanation, but the individual may have been in an aberrant physiological state or had abnormal food ingestion just before sampling.

### Community diversity of hornet gut microbiota

**Figure 3:**
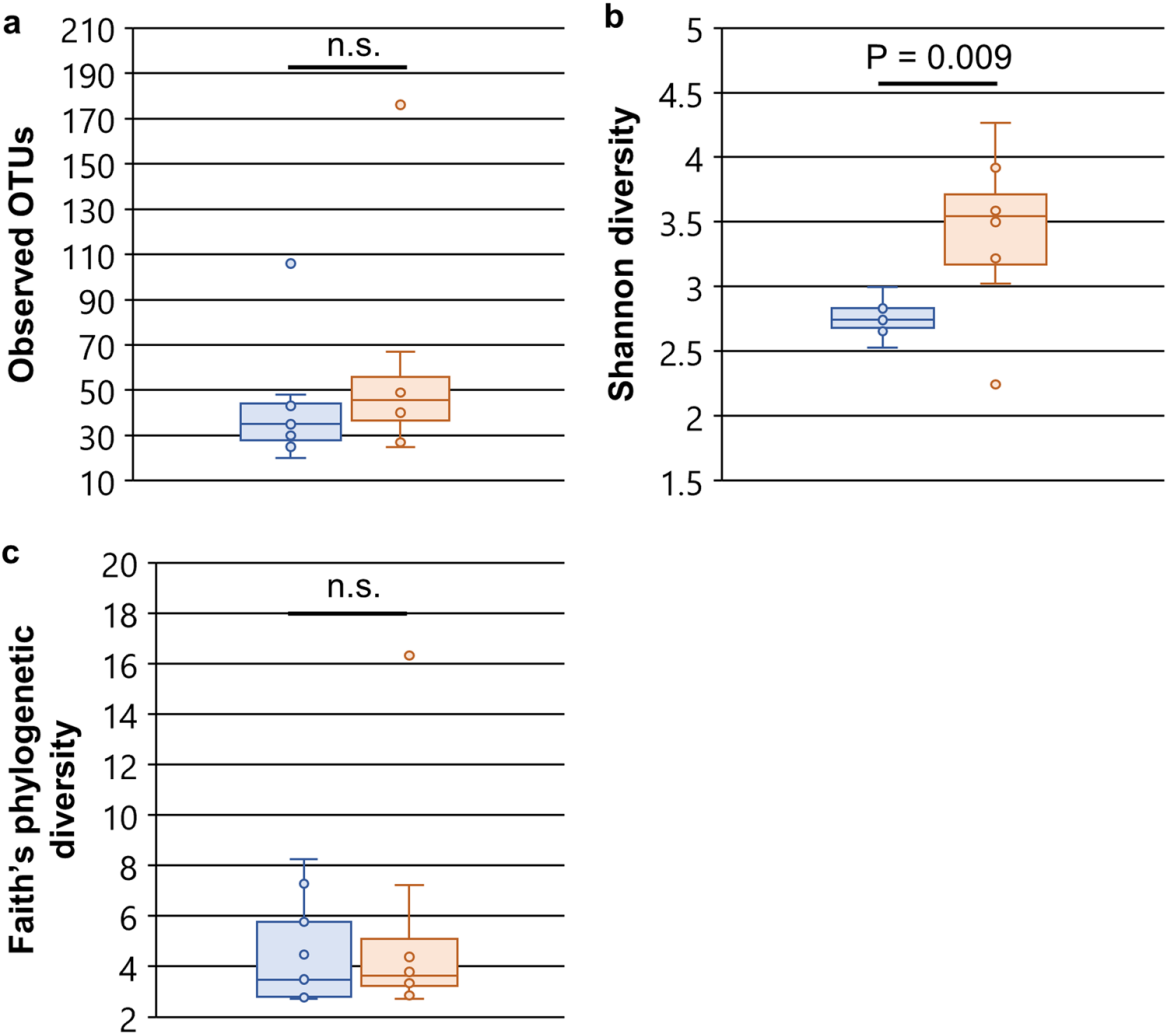
Comparison of alpha diversity metrics between hornet gut microbiomes. (**a**) Box-whisker plots of the numbers of observed OTUs, (**b**) Shannon diversity, and (**c**) Faith’s phylogenetic diversity. Points represent individual samples. Lines in the boxes correspond to the median of samples. Statistical significance was analyzed by Kruskal-Wallis test. n.s., not significant (P > 0.05). Blue, *V. mandarinia*; orange, *V. simillima*. *V. mandarinia*, N = 9; *V. simillima*, N = 8.

In order to statistically analyze the diversity of hornet gut microbial communities, we examined alpha and beta diversity metrics implemented in QIIME 2. While the average number of observed OTUs and Faith’s phylogenetic diversity for *V. mandarinia* and *V. simillima* were not significantly different (P > 0.05, Kruskal-Wallis test; Fig. 3a,c), *V*. *simillima* had a significantly higher Shannon diversity (P = 0.009, Kruskal-Wallis test; Fig. 3b). Thus, *V. mandarinia* and *V. simillima* have gut microbiomes that contain similar numbers of OTUs and are similarly diverged, but different in evenness.

**Figure 4:**
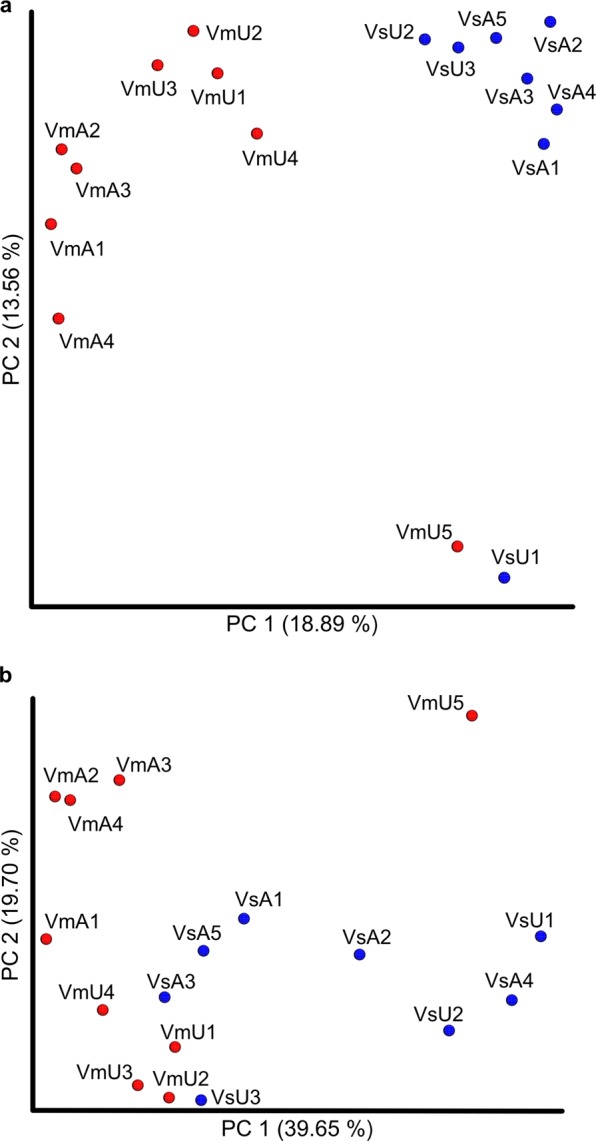
PCoA analysis of hornet gut microbiome samples. (**a**) PCoA plots of Jaccard distance. Each point represents individual sample and clustering of points means similarity in membership of OTUs among those samples. OTU membership was significantly different between host species (P = 0.0004, ANOSIM; P = 0.0002, PERMANOVA, 5000 times of permutations in each test). (**b**) PCoA plots of Bray-Curtis distance. Clustering of points means similarity in relative abundances of OTUs among those samples. Relative abundances of OTUs was significantly different between species (P = 0.007, ANOSIM and PERMANOVA, 5000 times of permutations in each test). Species and sampling locations were represented by sample IDs as follows; Vm and Vs, *V. mandarinia* and *V. simillima*, respectively; A and U, AIST and UT, respectively. For instance, VmA1 means *V. mandarinia* AIST-1. Red, *V. mandarinia*; blue, *V. simillima*.

Community dissimilarity between individuals was examined within and between species. The number of unique OTUs relative to the total number of OTUs between individuals was analyzed using Jaccard distance. Principle coordinate analysis of Jaccard distances clearly discriminated host species (Figs 4a and S2a) with statistical significance (P = 0.0004, ANOSIM; P = 0.0002, PERMANOVA, 5000 permutations in each test). Bray-Curtis distance also effectively discriminated host species (Figs 4b and S2b; P = 0.007, ANOSIM and PERMANOVA, 5000 permutations in each test). These analyses indicated that gut microbiome structure is distinguishable between hosts species. Interestingly, when the OTU phylogeny was considered, the differences between the hornet species was ambiguous: P > 0.05 for unweighted and weighted UniFrac (Fig S3; 5000 permutation tests of ANOSIM and PERMANOVA) but P = 0.02 for weighted UniFrac (5000 permutation tests of ANOSIM). However, when only the most dominant class Gammaproteobacteria was considered, UniFrac-based analyses showed a significant difference between hosts detected by both metrics (Fig. S4; unweighted UniFrac; P = 0.008, ANOSIM; P = 0.007, PERMANOVA. Weighted UniFrac; P = 0.02, ANOSIM and PERMANOVA. For all statistical tests, 5000 permutations were performed). This indicates that although phylogeny-based differences in whole gut microbiome structure was unclear, the hornet gammaproteobacterial community structure clearly differed phylogenetically.

When we examined the effect of sampling location on diversity indices, significant differences were neither detected for alpha-diversity (P > 0.05, Kruskal-Wallis test for each metric) nor beta-diversity metrics (P > 0.05, ANOSIM and PERMANOVA in 5000 permutation tests). There were only two exceptions: P = 0.03 and 0.02 for ANOSIM of Bray-Curtis distance and weighted UniFrac distance. Unweighted and weighted UniFrac distances of the Gammaproteobacteria communities were also not significantly different in any test (P > 0.05, ANOSIM and PERMANOVA in 5000 permutation tests). These results suggest that the sampling locations used in this study did not affect hornet gut microbiomes.

Results did not significantly change when the above analyses were repeated without *V. mandarinia* UT-5, an individual that had unique microbial community composition from other conspecifics (Fig. 2b; data not shown).

### Phylogenetic analysis of core OTUs in hornets

**Figure 5:**
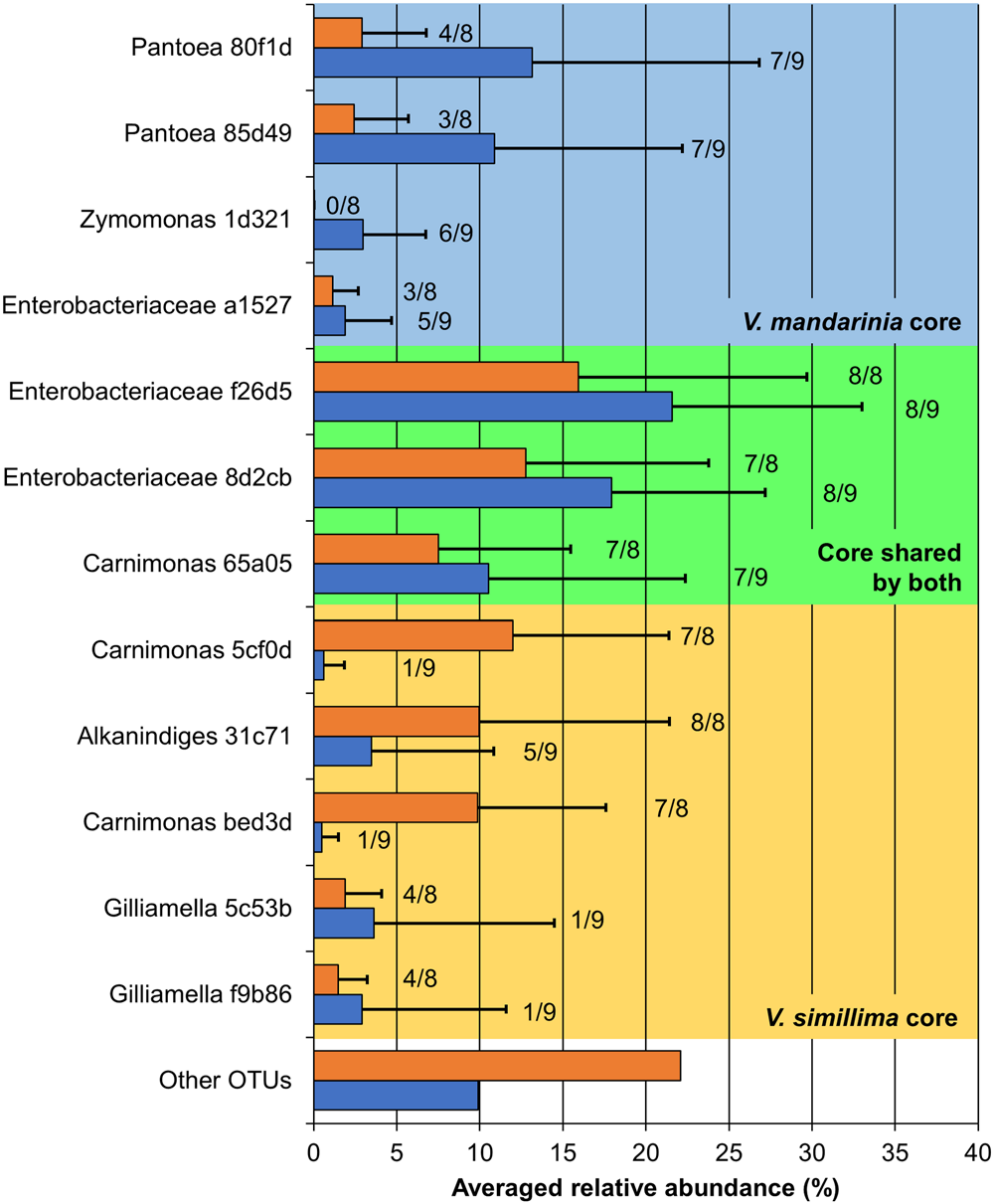
Relative abundance of core OTUs in *V. mandarinia* and *V. simillima*. When an OTU was detected in both locations and from at least 50% of samples at a relative abundance greater than 1%, it was defined as core. Bars indicate averaged relative abundances of core OTUs with standard deviations in *V. mandarinia* (blue) and *V. simillima* (orange). The ratio of samples containing each core OTU is shown on the right of each bar. The OTU IDs are shortened to the last five characters. Taxonomy of OTUs is assigned based on SILVA (also see Table 2). Bar colors; blue, *V. mandarinia*; orange, *V. simillima*. Core OTUs unique to *V. mandarinia*, unique to *V. simillima*, and shared by both are highlighted in blue, yellow, and green, respectively.

To understand the symbiotic relationship between the host hornet and its gut microbiota, we investigated whether these hornets have core gut microbial populations. In this study, we defined core populations (i.e., OTUs) as those found in hosts from both locations and present in at least half of the individuals (*V. mandarinia* or *V. simillima*) at a relative abundance greater than 1%^20^. Intriguingly, despite the low community dissimilarity among individuals of each hornet species, only a small number of core OTUs was identified in each hornet species (seven and eight OTUs for *V. mandarinia* and *V. simillima* respectively; Fig. 5). Among the core OTUs, only three were shared between the *Vespa* species (two Enterobacteriaceae and one *Carnimonas*; SILVA-based annotation) and the remaining were only defined as core in *V. mandarinia* or *V. simillima* (four and five OTUs respectively). The *V. mandarinia*-associated core OTUs were assigned to *Pantoea* (Enterobacteriales), *Zymomonas* (Sphingomonadales), and Enterobacteriaceae (Enterobacteriales). Those associated with *V. simillima* belonged to *Carnimonas* (Oceanospirillales), *Alkanindiges* (Pseudomonadales), and two *Gilliamella* (Orbales). Using other 16S rRNA gene databases, Greengenes and EzTaxon, resulted in similar classification with SILVA at higher taxonomic levels but we observed slight differences at lower levels (Table 2), possibly due to lack of recent updates in Greengenes and different clustering cutoff (97% identity) in EzTaxon. We also found that close relatives of the core OTUs (≥98% nucleotide sequence identity based on a BLAST search against the nr/nt database) had similar taxonomic classification to those OTUs (Table S1). Most of these sequences were associated with samples from plants and insects based on metadata available on NCBI Genbank (Table S1), implying that the hornet core OTUs might have originated from such sources.

**Table 2:** Taxonomic classification of core OTUs in hornet gut microbiome based on different 16S rRNA gene databases.

Host	OTU	SILVA	Confi-dence	Greengenes	Confi-dence	Eztaxon	Confi-dence
*V. mandarinia*	80f1d	Enterobacteriaceae; Pantoea	0.818	Enterobacteriaceae	1	Enterobacteriaceae	1
	85d49	Enterobacteriaceae; Pantoea	0.802	Enterobacteriaceae	1	Enterobacteriaceae	1
	1d321	Sphingomonadaceae; Zymomonas; Ambiguous taxa	0.852	Sphingomonadaceae; Sphingomonas; Sphingomonas wittichii	1	Sphingomonadaceae; Zymomonas; Zymomonas mobilis	1
	a1527	Enterobacteriaceae	1	Enterobacteriaceae	1	Enterobacteriaceae; Salmonella; Salmonella enterica	0.857
*V. mandarinia*/*V. simillima*	f26d5	Enterobacteriaceae	1	Enterobacteriaceae	1	Enterobacteriaceae; Lonsdalea; Lonsdalea quercina	0.999
	8d2cb	Enterobacteriaceae	1	Enterobacteriaceae	1	Enterobacteriaceae; Lonsdalea; Lonsdalea quercina	0.999
	65a05	Halomonadaceae; Carnimonas; uncultured bacterium	0.962	Halomonadaceae	0.93	Halomonadaceae; Zymobacter; Zymobacter palmae	0.972
*V. simillima*	5cf0d	Halomonadaceae; Carnimonas; uncultured bacterium	0.746	Halomonadaceae	0.82	Halomonadaceae	0.994
	31c71	Moraxellaceae; Alkanindiges; uncultured bacterium	0.721	Moraxellaceae; Acinetobacter	0.821	Moraxellaceae	0.999
	bed3d	Halomonadaceae; Carnimonas; uncultured bacterium	0.722	Halomonadaceae	0.804	Halomonadaceae	0.994
	5c53b	Orbales; Orbaceae; Gilliamella; uncultured gamma proteobacterium	0.903	Pasteurellales	1	Orbales; Orbaceae; Gilliamella	0.99
	f9b86	Orbales; Orbaceae; Gilliamella; uncultured gamma proteobacterium	0.908	Pasteurellales	1	Orbales; Orbaceae; Gilliamella	0.99

Classification of core OTUs using SILVA, Greengenes, and EzTaxon, are shown at deeper levels than the class. Confidence is probability that the OTU was assigned to the represented taxon. The OTU IDs were shortened to the last five characters.

**Figure 6:**
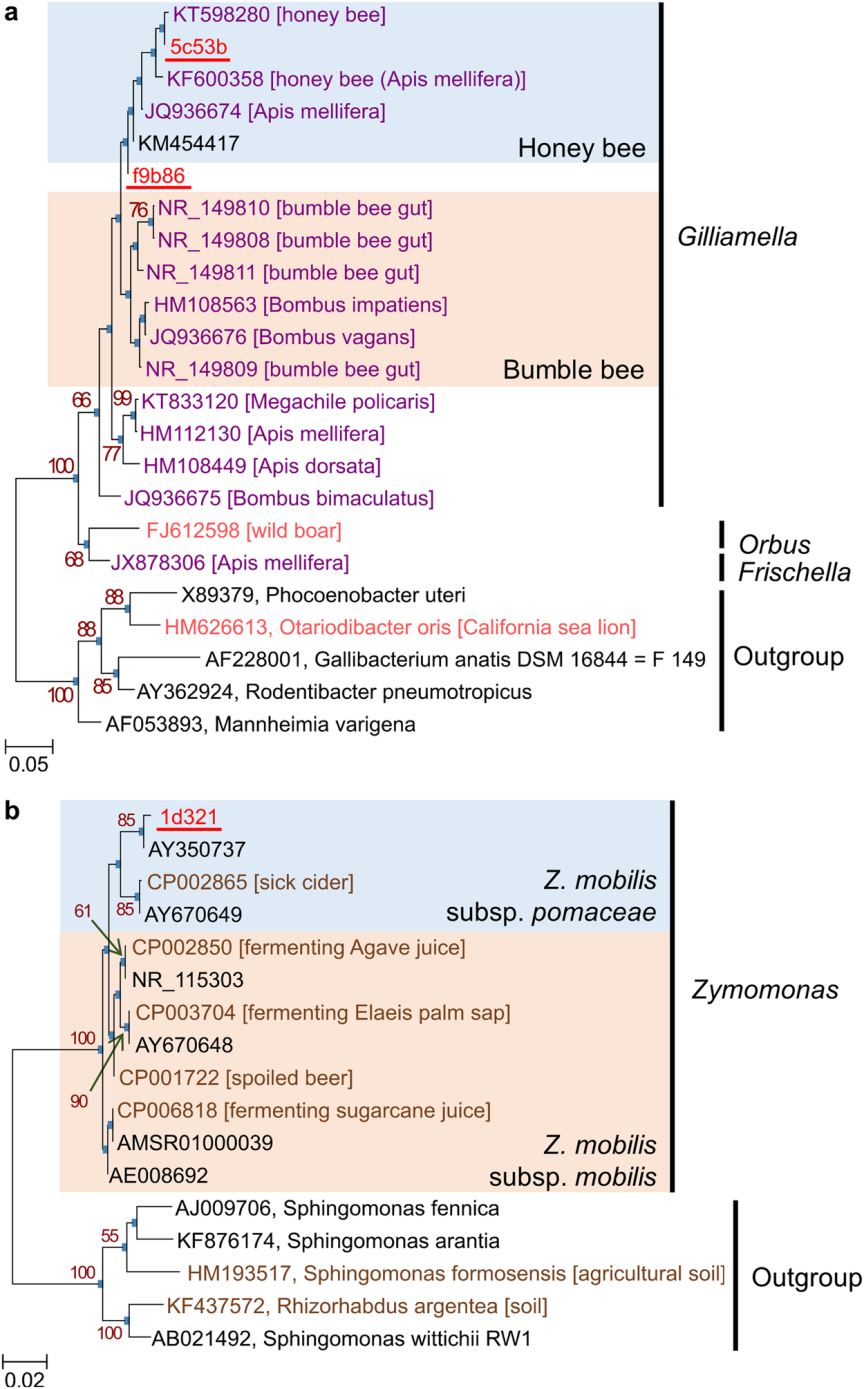
Phylogenetic analysis of *Gilliamella*- and *Zymomonas*-related core OTUs in hornet guts. Maximum-likelihood trees of (**a**) *Gilliamella* and (**b**) *Zymomonas*. Phylogenetic analyses were performed with maximum-likelihood method. Bootstrap values > 50% are shown. OTUs detected in hornets are red and underlined. Isolation site or host animals, for which Genbank database in NCBI was referred, are indicated in brackets. Scale bar indicates substitutions per position. Accession numbers are colored depending on isolation sites as follows: vermilion, mammal; purple, insect; brown, others; black, not described.

To improve the phylogenetic classification of core populations, we performed phylogenetic analysis of the core OTUs using the SILVA database and ARB^21,22^ or RAxML^23^. Core *Gilliamella* OTUs of *V. simillima* (5c53b and f9b86) were closely related to a sublineage of *Gilliamella* strains detected in the honey bee *Apis mellifera* (99.3–100% sequence similarity for both OTUs; Figs 6a and S5a). Given that *Gilliamella* is a unique microbe in eusocial corbiculate bees^10^, suggesting that the core *Gilliamella* population could be derived from predated honey bees. The *Zymomonas*-related core OTU of *V. mandarinia* (1d321) closely related with *Z. mobilis*, an organism associated with fermenting plant sap or spoiled alcohol^24^ (Figs 6b and S5b). Both maximum-likelihood and maximum-parsimony trees suggest that 1d321 belongs to a sublineage of *Z. mobilis* subsp. pomaceae. A core *Enterobacteriaceae* OTU of *V. mandarinia* (a1527) was found at the stem of a clade consisting of *Tatumella*, *Phaseolibacter* and *Rosenbergiella* (Fig. S6). *Enterobacteriaceae* OTUs f26d5 and 8d2cb, shared by both hornets, were clearly positioned into clades of *Yersinia* (Fig. S7) and *Lonsdalea* (Fig. S8), respectively. Positions of other core OTUs were inconsistent with classification in QIIME 2. For example, while 80f1d and 85d49 were annotated as *Pantoea* in QIIME 2, ARB-based analysis revealed that they were rather related to *Gibbsiella* (Fig. S7) and *Enterobacillus* (Fig. S9), respectively. Similarly, 65a05, 5cf0d, and bed3d were determined to be related to *Zymobacter palmae* (Fig. S10), an uncharacterized *Zymobacter*-related lineage of *Halomonadaceae*, and 31c71 was an uncharacterized *Acinetobacter*-related lineage of *Moraxellaceae* (Fig. S11).

### Honey bee gut microorganisms in hornets

**Figure 7:**
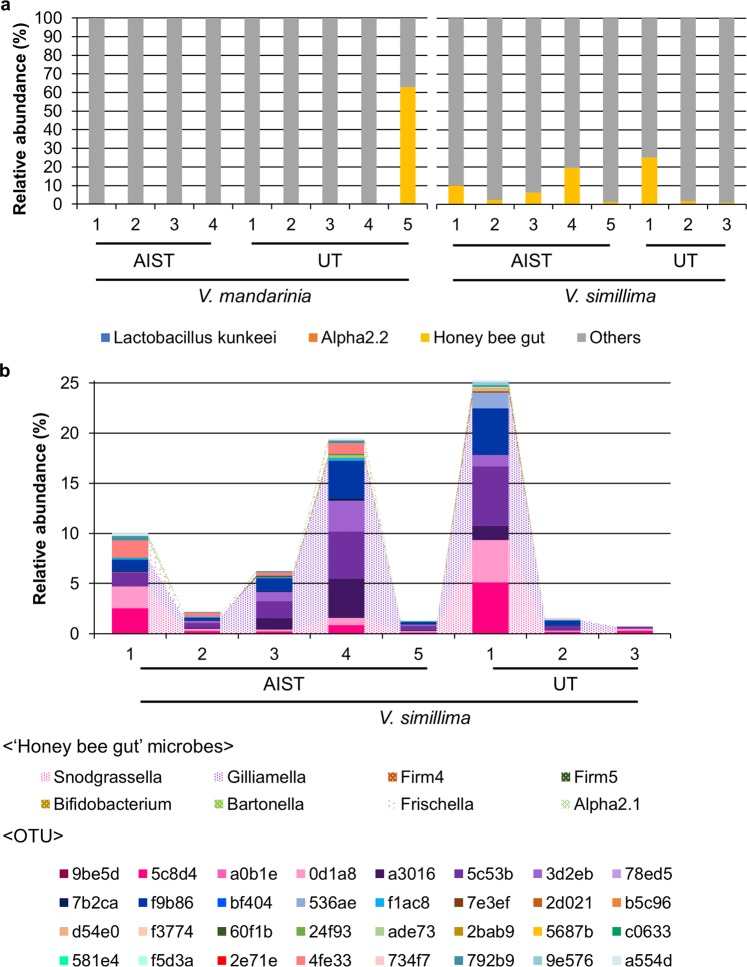
Honey bee gut microbiome in hornets. (**a**) Relative abundance of OTUs annotated as microbes associating honey bee crop (*Lactobacillus kunkeei*, blue; Acetobacteraceae Alpha2.2, orange), populations abundant in honey bee gut (represented as “honey bee gut”, yellow), and others (grey) in hornet gut microbiome. “Honey bee gut” contains *Snodgrassella*, *Gilliamella*, *Lactobacilli* (Firm4 and Firm5), *Bifidobacterium*, *Bartonella*, *Frischella*, and Acetobacteraceae Alpha2.1. *L. kunkeei* was detected only in *V. mandarinia* UT-5. Alpha2.2 was detected only in *V. mandarinia* UT-1, *V. simillima* AIST-1, AIST-2, and AIST-4. “Honey bee gut” was detected in all samples except for *V. mandarinia* AIST-2, UT-1, UT-2, and UT-3. (**b**) Relative abundance of “honey bee gut” microbes in *V. simillima* at the genus and OTU levels. Genus is represented by area chart, while OTU is shown by bar plots. The scale is same as used in (**a**). The OTU IDs were shortened to the last five characters. AIST, the National Institute of Advanced Industrial Science and Technology; UT, the University of Tokyo.

Given that two core OTUs of *V. simillima* are *Gilliamella* (5c53b and f9b86), a genus of characteristic of the honey bee gut^10,11^, we suspected that honey bee-associated gut microbes could migrate to and colonize the hornet gut. To test this hypothesis, we here focused on 76 OTUs which were annotated in QIIME 2 (based on SILVA) as *Snodgrassella*, *Gilliamella*, *Lactobacillus*, *Bifidobacterium*, *Bartonella*, or Acetobacteraceae, known to be abundant bacteria in the honey bee gut or crop^10,25^. Similar taxonomic classification was annotated with closest relatives (≥97% sequence identity to those OTUs) found in BLAST search, except that some OTUs annotated as *Gilliamella* in QIIME 2 were similar to *Frischella*. Relative abundance of those OTUs similar to honey bee gut/crop bacteria varied among hornets (Fig. 7a). OTUs similar to crop-dominating bacteria (*Lactobacillus kunkeei* and Acetobacteraceae Alpha2.2)^25^ were detected in less than half of the individuals with less than 0.05% relative abundance (*V. mandarinia* UT-1 and UT-5; *V. simillima* AIST-1, AIST-2, and AIST-4), suggesting that they are not stably maintained in hornet guts (Fig. 7a). OTUs related to abundant species in the honey bee gut, *Snodgrassella*, *Gilliamella*, *Lactobacillus* Firm4 and Firm5, *Bifidobacterium*, *Bartonella*, *Frischella*, and Acetobacteraceae Alpha2.1^10^, were sporadic in *V. mandarinia*; they were detected with less than 0.5% relative abundance in four out of nine samples (AIST-1, AIST-3, AIST-4, and UT-4) and abundant (62.9%) in UT-5 (Fig. 7a). In contrast, all *V. simillima* samples harbored those OTUs at more than 1% relative abundance, except UT-3 exhibiting 0.72% (Fig. 7a). These results suggest that honey bee gut microbes are consistently found in the gut of *V. simillima* but not *V. mandarinia*. The honey bee-related populations in *V. simillima* comprised 5–20 OTUs (average 11.9 ± 5.11 OTUs per sample) and were classified into 3–8 genera (Fig. 7b). The composition was relatively similar among *V. simillima* individuals, and OTUs belonging to *Snodgrassella* and *Gilliamella* were prominent (Fig. 7b). Notably, this was quite different from the general honey bee gut microbiota composition (Fig. S12) in which *Lactobacillus* (Firm4 and Firm5), *Bifidobacterium*, and *Bartonella* were more abundant^10^.

## Discussion

This is the first comprehensive characterization of gut microbiomes in hornets, *V. mandarinia* and *V. simillima*. While our study detected a total of 465 OTUs, only seven and eight core OTUs were found in *V. mandarinia* and *V. simillima*, respectively (Fig. 5). Previous studies have also reported simple gut microbiomes in social bees, such as the honey bee, stingless bee, bumble bee (five OTUs of core bacteria)^10,11^, and a facultatively eusocial bee *Megalopta* (two OTUs)^20^. As with these bees that have specific food habit (i.e., nectar and pollen)^26^, adult hornets depend on liquid foods such as plant saps and larval saliva, so the small number of core OTUs may have some relation to such food habit as well as social behavior. Apparently, the gut community in *V. mandarinia* was more consistent and uniform than that in *V. simillima* (Fig. 2b), which might be related to the different feeding preference of the hosts – *V. simillima* is more generalist than *V. mandarinia*.

Many core OTUs are closely related to organisms possibly associated with potential *Vespa* carbohydrate sources (Table S1), such as tree sap, flower nectar, and ripe fruit. *Gibbsiella* and *Lonsdalea* relatives (80f1d and 8d2cb respectively; Figs S7 and S8) found in *V. mandarinia* are often isolated from oak^27^, possibly reflecting the tendency of *V. mandarinia* to prefer oak sap^12,13^. Another specific core OTU, 1d321, in *V. mandarinia* is a close relative of *Zymomonas mobilis*, which are often associated fermenting plant sap (e.g., palm, Agave, sugarcane) (Figs 6b and S5b) or spoiled alcoholic liquids^24^. Given that such fermentation odor and plant sap attracts hornets^17,28^, *Z. mobilis* may also be acquired through diet. *Z. mobilis* can fix nitrogen^29^ and this could potentially serve *V. mandarinia* as an alternative nitrogen source to amino acid-containing larval saliva^13,30^. A unique core OTU, 31c71, in *V. simillima* is related to *Acinetobacter* species isolated from plants or insects (Fig. S11), implying some relation between this OTU and the diet of *V. simillima*.

Other core OTUs are closely related to microorganisms associated with honey bees. A *Zymobacter palmae* relative (OTU 65a05) found in both hornets is closely related to sequences detected in processed pollen in honey bee hives^31^, suggesting acquisition of gut microorganisms from contact with prey honey bees. Two other core OTUs unique to *V. simillima* (5c53b and f9b86) relate to a gastrointestinal *Gilliamella apicola* clade dominant in European honey bees (*Apis mellifera*), a prey species of hornets (Figs 6a and S5a). Further analysis revealed that other OTUs closely related to honey bee gut microbes (e.g., *Snodgrassella*, *Gilliamella*, and *Frischella*) were detected in almost all *V. simillima* samples at >1% relative abundance (Fig. 7a). Even populations rare in the honey bee gut (*Frischella*-, *Bartonella*-, and Alpha2.1-related OTUs) are detected in *V. simillima* samples, albeit sparsely. Intriguingly, other dominant core bacteria in the honey bee gut, *Lactobacillus* and *Bifidobacterium*, are rare in hornet guts (Fig. S12). We currently hypothesize that the hornets do not require those species because they may already have alternative fermenters (i.e., *Zymomonas* 1d321 and *Zymobacte*r 65a05). Overall, this suggests that the *V. simillima* gut is exposed to honey bee-derived microbial populations and likely selects for specific microbial groups (perhaps Gram negative). To further highlight the selectivity of this process, *V. mandarinia* only sparsely possesses microbial populations associated with their prey at low abundance (e.g., Coleoptera, or honey bees), or select organisms detected in another prey in this study (*V. simillima*).

The two hornet species interestingly share one enigmatic core OTU f26d5 related to *Yersinia* (Fig. S7). *Yersinia* is known for pathogenicity and the closest relative (*Y. ruckeri*) is also characterized as a fish pathogen^32,33^. However, considering the lifestyles of *V. mandarinia* and *V. simillima*, acquisition of f26d5 from fish is unlikely. As f26d5 is the most dominant OTU (15.9–21.6%) and detected in almost all samples (Fig. 5), the microbe may play an important but unknown role in the host biology.

In the present study, we uncovered gut microbiota of two hornet species. They had small sets of core OTUs (i.e., seven or eight) with host-specific differences in the number of unique OTUs and composition. Most core OTUs have close relatives that can be associated with their food source. Hence, our current hypothesis is that the core OTUs are directly related to the hornet diet and have not undergone extensive coevolution with the host, unlike those of corbiculate bees^10,11^. Moreover, the gut of the *V. mandarinia* and *V. simillima* appear to preferentially harbor bacteria respectively originated from their carbon source and prey. However, as the biological function and origins of some core OTUs have not been addressed, further studies on the association between hornets and gut microbiota is necessary to elucidate how gut microbiota influence hornet biology. Comparison of these insights with knowledge obtained in other social insects will deepen our understanding on relationship between gut microbiota and sociality or food habit of insects.

## Methods

### Sample collection

*V. mandarinia* and *V. simillima* adults were collected at the National Institute of Advanced Industrial Science and Technology (AIST; N36°03′48.1″ and E140°07′54.1″), and the University of Tokyo (UT; N35°42′35.9″ and E139°45′39.8″) in Japan in Fall of 2017. The adults which came to the hives of honey bee were randomly caught using an insect net. They were transferred into a 50- or 15-mL plastic conical tube, anesthetized on ice, and immersed in absolute ethanol and stored at −20 °C until use. Tubes containing samples were put on dry ice when samples were transferred from UT to AIST.

### Dissection and DNA extraction

Before dissection, tweezers were sterilized with 70% ethanol and UV light irradiation for 10 min. Insects were dissected under a binocular microscope. The gut was roughly homogenized using tweezers in 978 µL of sodium phosphate buffer included in FastDNA Spin Kit for Soil (MP-Biomedicals), and transferred into Lysing Matrix E (MP-Biomedicals). DNA extraction was performed according to the manufacturer’s instruction with beads-beating for 1 sec at 5,500 rpm, using Micro Smash MS-100 (TOMY). For *V. mandarinia* samples collected at AIST, beads-beating was performed twice. Extracted DNA samples were quantified using Qubit dsDNA HS Assay Kit (Molecular Probes), and their integrity were verified by agarose electrophoresis.

### 16S rRNA gene amplification and sequencing

The V4 region of the bacterial 16S rRNA gene was amplified using a 2 ng of DNA template and primers 515 F (5′-GTGCCAGCMGCCGCGGTAA-3′) and 806R (5′-GGACTACHVGGGTWTCTAAT-3′). PCR reactions were performed in 20 μl with ExTaq HS (TaKaRa) at an annealing temperature of 50 °C for following cycles; 23 and 27 cycles for *V. mandarinia* collected AIST and UT, respectively; 25 cycles for *V. simillima*. These cycles where amplifications were not saturated were determined by semi-quantitative PCR. The PCR products were purified using AMPure XP beads (Beckman Coulter) and eluted with 20 μl of distilled water. A 2 μl of each eluate was used for subsequent short PCR with Illumina barcoded primers for 8 cycles. The PCR products were purified with AMPure XP beads and eluted with 22 μl of distilled water. The amplicon library was sequenced by Illumina MiSeq 2 × 250 bp pair-end platform.

### 16S rRNA-based community analysis

Read data was analyzed using QIIME 2 (https://qiime2.org)^34^, according to online manuals (https://docs.qiime2.org/2018.6/tutorials/moving-pictures/). Paired-end sequences imported into QIIME 2 were quality-controlled and combined using DADA2 (–p-trunc-len-f 175–p-trunc-len-r 144–p-trunc-q 20–p-trim-left-f 13–p-trim-left-r 13)^35^. The settings for quality control was based on the reads’ quality distribution along the length of the sequence. This grouped sequences into operational taxonomic units (OTUs) based on 100% sequence similarity. Alpha rarefaction analysis, taxonomic classification of OTUs, alpha diversity (the number of observed OTUs, Shannon diversity, and Faith’s phylogenetic diversity), and beta diversity (Jaccard distance, Bray-Curtis distance, unweighted and weighted UniFrac distances) were analyzed using QIIME 2. Alpha rarefaction curve was plotted with 50 sampling depths. For taxonomic classification, Greengenes 13_8 99% OTUs (Greengenes)^36^, SILVA 132 99% OTUs (SILVA)^18,19^, and EzTaxon 97% OTUs^37^ were utilized as 16S rRNA gene databases. Taxonomic classifier implemented in QIIME 2 was originally trained by 515F/806R region of Greengenes-registered sequences. When SILVA and EzTaxon were used, sequences between 515F and 806R region were extracted from databases to train the taxonomic classifier (feature-classifier “extract-reads” and “fit-classifier-naive-bayes”). Statistical analyses for diversity metrics and generation of principal coordination analysis (PCoA) plots for beta diversity metrics were also done through QIIME 2 (diversity “core-metrics-phylogenetic”, “alpha-group-significance”, and “beta-group-significance”). Sampling depth (–p-sampling-depth) was set to 12627. To analyze unweighted and weighted UniFrac distances of Gammaproteobacteria OTUs, the dataset was filtered to obtain only OTUs annotated as Gammaproteobacteria based on SILVA (taxa “filter-table” and “filter-seqs”). Then, alignment of remaining OTU sequences (alignment “mafft” and “mask”), phylogenetic tree construction (phylogeny “fasttree” and “midpoint-root”), and core-metric analyses were performed. Sampling depth in core metric analyses (–p-sampling-depth) was 11,695, which was the minimum number of remaining read counts among samples.

### Detailed phylogenetic analysis of core OTUs

Phylogenetic trees were constructed to confirm and/or improve the phylogenetic annotation of the core OTUs. Nucleotide sequences of core OTUs and their relatives in the NCBI Nucleotide database were aligned using SINA version 1.2.11 with the default setting^22^, which aligns input sequences with sequences registered in SILVA (version 132). Based on the alignment, phylogenetic trees were constructed using SILVA in ARB software version 5.5^21^ by maximum parsimony method with ssuref:Bacteria filter. Additional relatives of the OTUs were selected in ARB to construct trees for individual lineages of interest: sequences with a length of at least 1266 bp were used to construct trees through the neighbor-joining method (ssuref:Bacteria filter with Jukes-Cantor correction and bootstrap number of 1000), and shorter sequences (e.g., sequences obtained in this study) were inserted through the maximum-parsimony method (ssuref:Bacteria filter). Phylogenetic trees with maximum-likelihood method were also constructed for *Zymomonas* and *Gilliamella* bacteria. Their sequences were aligned through SINA (with the default setting) and processed through RAxML^23^ with GTR + G model and 200 times of bootstrap. The resulting trees were visualized using ETE2^38^. Online BLAST search was performed to obtain further information on close relatives of OTUs.

## Supplementary information

Supplementary information

## Data Availability

The 16SrRNA gene amplicon datasets generated during this study are deposited and available in the Sequence Read Archives of National Center for Biotechnology Information (NCBI), European Bioinformatics Institute (EBI) and DNA Data Bank of Japan (DDBJ), http://trace.ddbj.nig.ac.jp/DRASearch, under accession number DRA007725.
