# Supplementary material for: Community analysis of gut microbiota in hornets, the largest eusocial wasps, Vespa mandarinia and V. simillima
Source: Sci Rep. 2019 Jul 8;9:9830. doi: 10.1038/s41598-019-46388-1 (PMC6614390; doi:10.1038/s41598-019-46388-1)
Supplement: Supplementary file 1 — Supplementary information [file 41598_2019_46388_MOESM1_ESM.pdf]

Supplementary information to

Community analysis of gut microbiota in hornets, the largest eusocial wasps, *Vespa mandarinia* and *V. simillima*

Shota Suenami, Masaru Konishi Nobu, and Ryo Miyazaki

* Corresponding author
Email: ryo.miyazaki@aist.go.jp

Supplementary Figure S1-S12
Supplementary Table S1

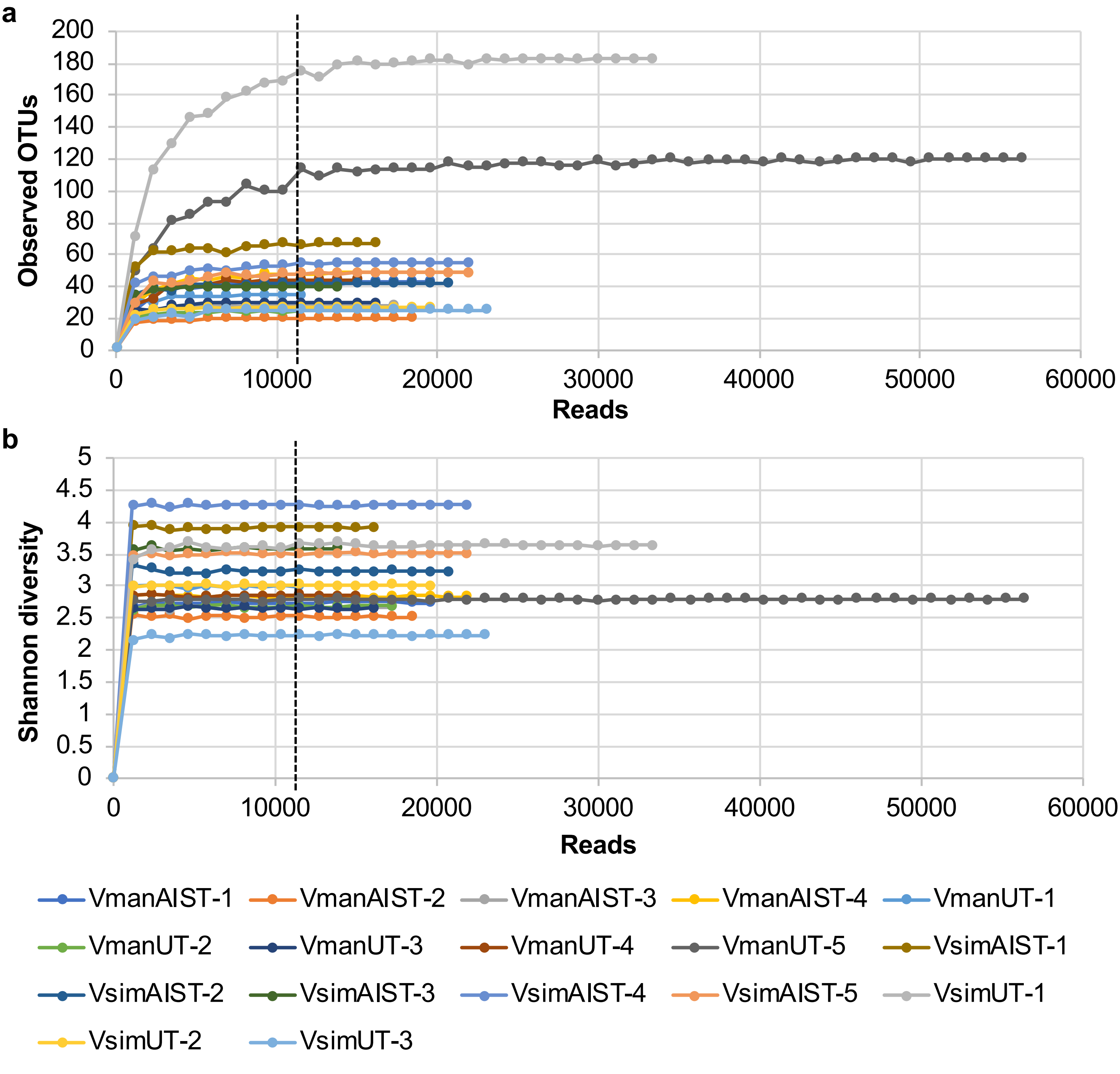

Fig S1. Alpha rarefaction curves of hornet gut microbiomes. The numbers of observed OTUs (a) and Shannon diversity (b) were plotted against read counts. Rarefaction curves were plotted at 50 sampling depths without iteration. Dashed lines indicate 11502 reads, which is the highest read count including all samples in this plot. Note that most samples reached plateau at this depth. In other analyses, we used 12627 reads for each sample. Sample IDs of *V. mandarinia* and *V. simillima* were shortened to Vman and Vsim, respectively. For instance, VmanAIST-1 represents *V. mandarinia* AIST-1.

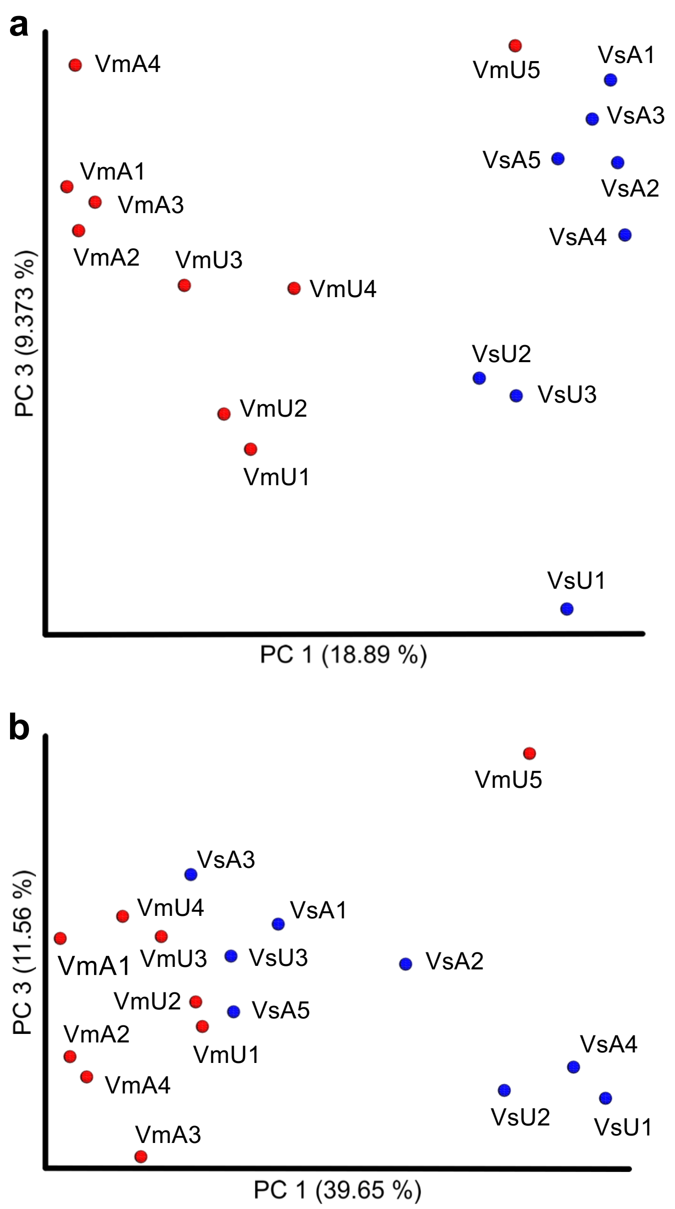

Fig S2. PCoA plots of hornet gut microbiomes based on Jaccard distance and Bray-Curtis distance observed from the axes 2. (a) Jaccard distance and (b) Bray-Curtis distance. PCoA plots of Fig 4 were rotated longitudinally around the axes 1. Clustering of host species was maintained for both distances. See Fig 4 for plots.

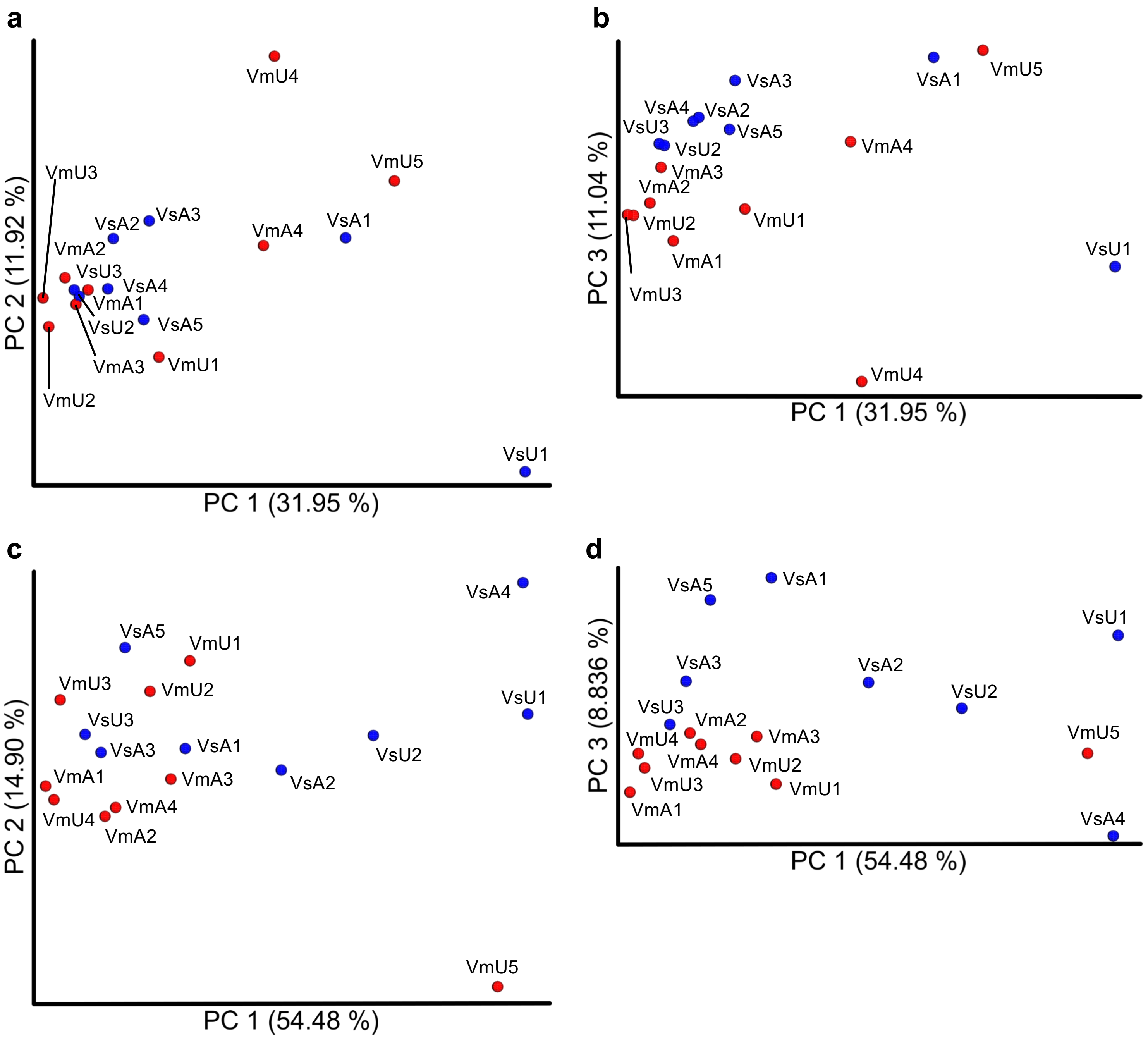

Fig S3. PCoA plot of hornet gut microbiomes based on unweighted or weighted UniFrac. (a and b) Unweighted UniFrac and (c and d) weighted UniFrac. Plots were viewed from the axis 3 (a and c) or axis 2 (b and d). Each point represents individual. Note that points with different colors (i.e. different host species) are not clearly separated ($P > 0.05$, ANOSIM and PERMANOVA in 5000 permutations for each test) while ANOSIM detected significant difference in weighted UniFrac ($P = 0.02$ in 5000 permutations). See Fig 4 for plots.

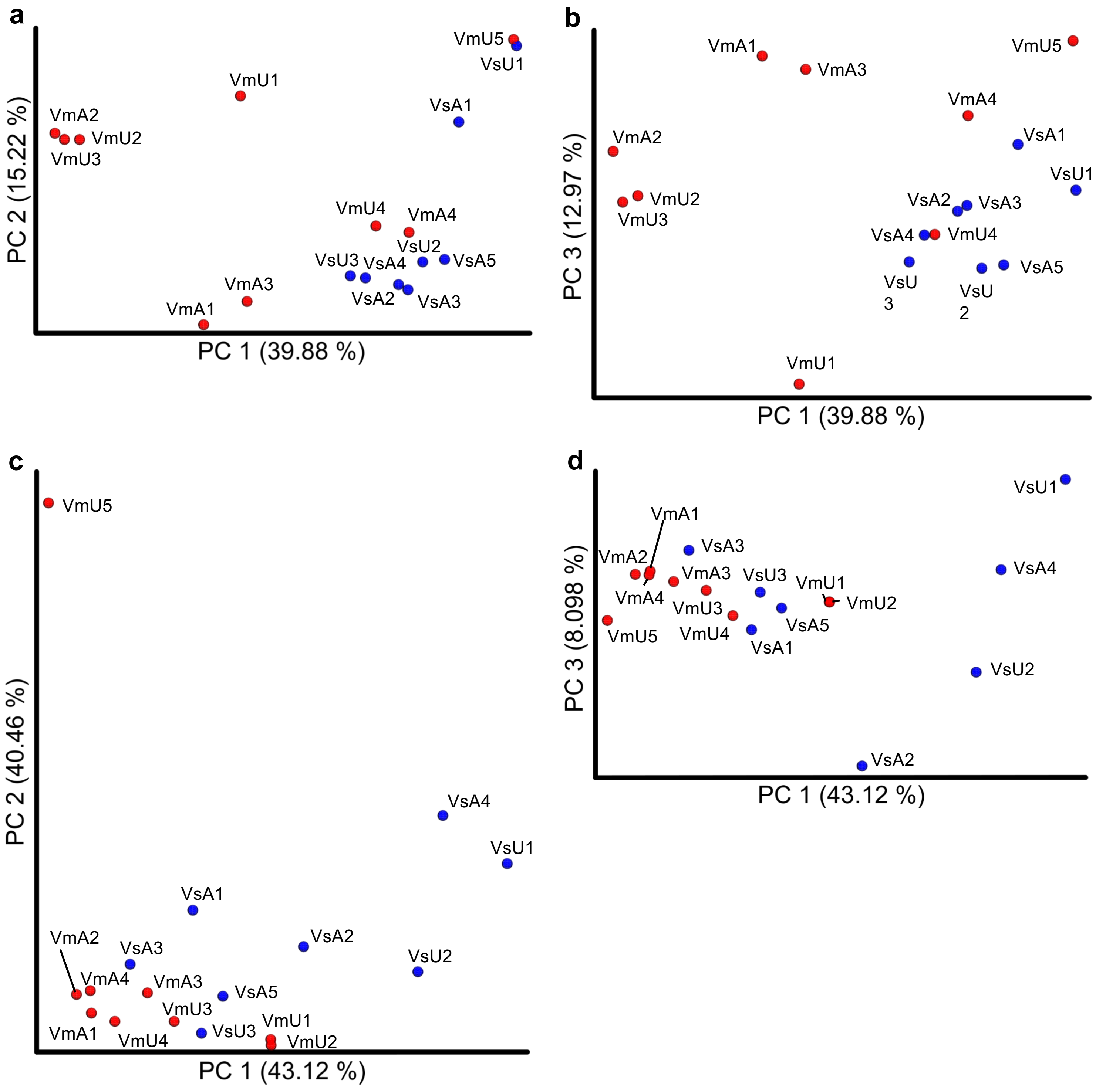

Fig S4. PCoA plot of hornet gut microbiomes restricted to Gammaproteobacteria on unweighted or weighted UniFrac. (a and b) unweighted UniFrac and (c and d) weighted UniFrac. Plot was viewed from the axis 3 (a and b) or axis 2 (b and f). Each point represents individual. Note that points representing *V. simillima* clustered and this cluster weakly separated from points of *V. mandarinia* (P = 0.008 and 0.007 for unweighted UniFrac; P = 0.02 for weighted UniFrac, ANOSIM and PERMANOVA respectively, in 5000 permutations for each test). See Fig 4 for plots.

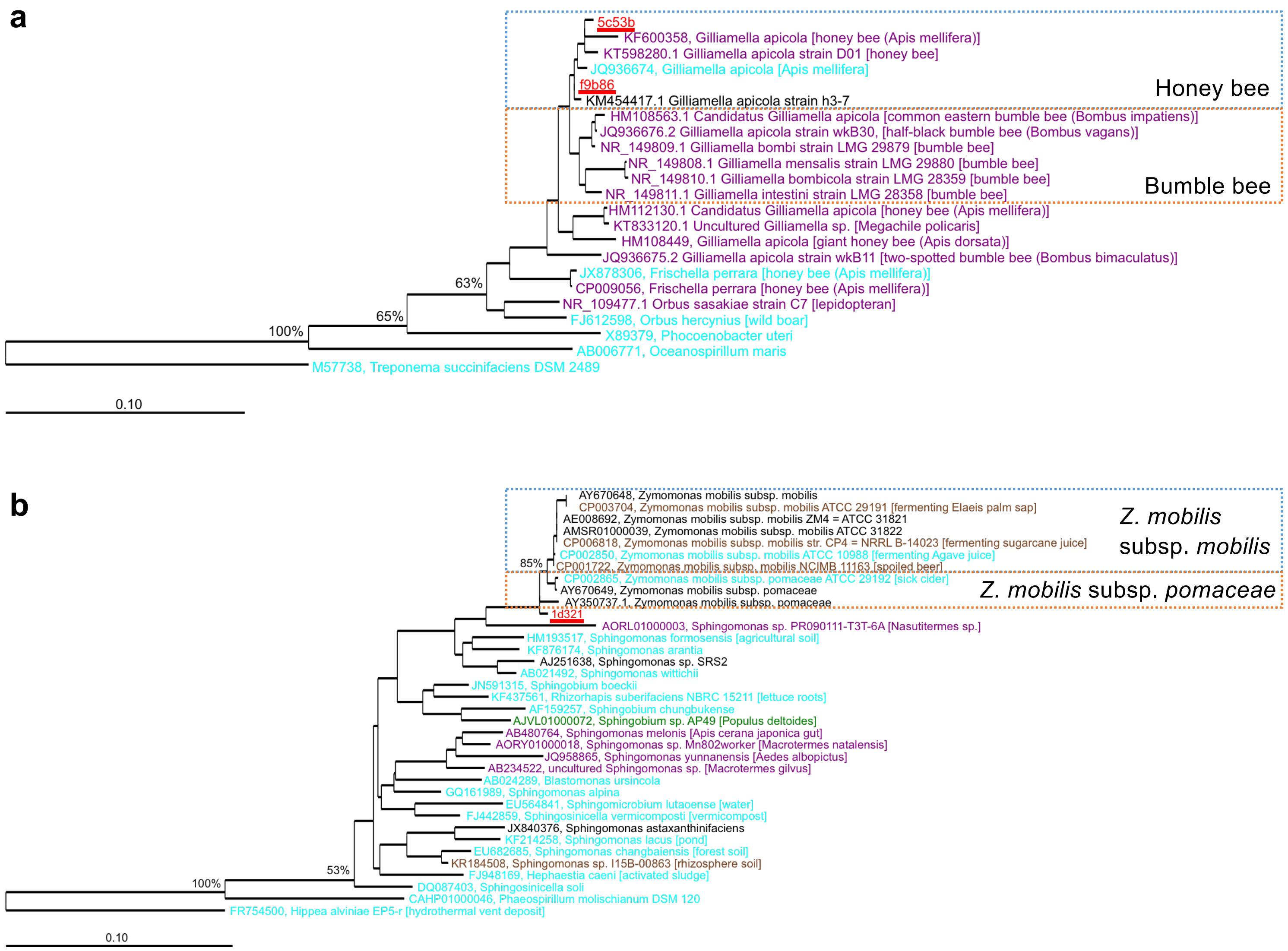

Fig S5. Phylogenetic trees of 5c53b, f9b86, and 1d321. Maximum-parsimony trees containing (a) 5c53b and f9b86 and (b) 1d321. Accession numbers and names of relatives are shown. Isolation sources, referred to SILVA or NCBI Nucleotide databases, are shown in brackets. Values at nodes are bootstrap values in 1000 times when small trees were constructed. Bootstrap values < 50 % are omitted. Scale bar indicates substitutions per position. *Gilliamella* clades detected in the honey bees and bumble bees are squared in blue and orange, respectively. Subspecies of *Zymomonas* were squared (*Z. mobilis* subsp. *mobilis* in blue and subsp. *pomaceae* in orange). M57738 and FR754500 were included as outgroups in (a) and (b), respectively. OTUs detected in our analysis are represented in red and underlined. Names in cyan are isolated strains, which were used in construction of small trees. Other sequences are shown in colors representing isolated sources as followings; green, plant; purple, invertebrate; brown, other; black, not descripted.

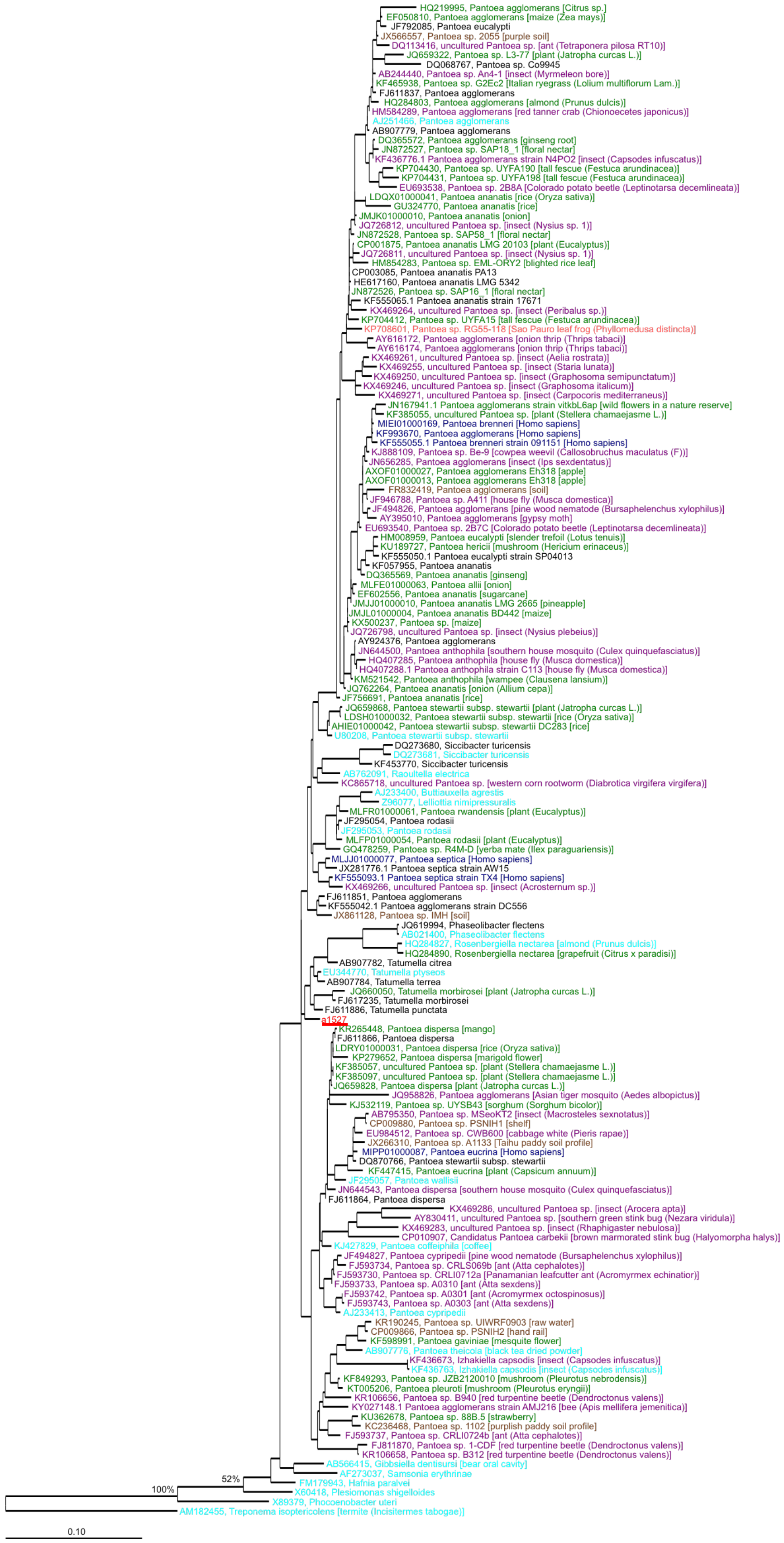

Fig S6. Phylogenetic tree containing a1527 and relative Enterobacteriaceae. AM182455 was used as outgroup. The OTU detected in our analysis is represented in red and underlined. Names in cyan are isolated strains, which were used in construction of small trees. Other sequences are shown in colors representing isolated sources as followings; green, plant; vermilion, vertebrate; purple, invertebrate; navy, human; brown, other; black, not descripted. See Fig S5 for other information.

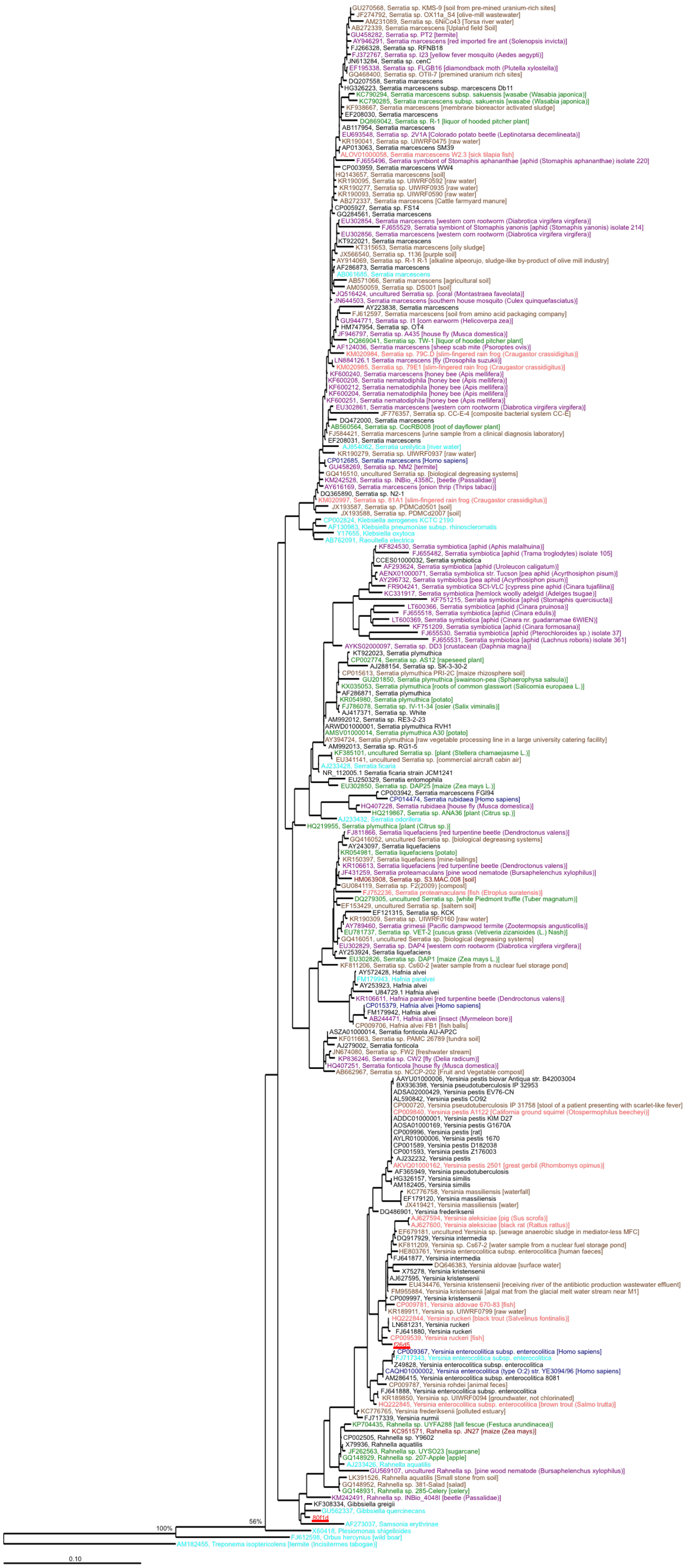

Fig S7. Phylogenetic tree containing 80f1d, f26d5 and relative Enterobacteriaceae. Names in cyan are isolated strains, which were used in construction of small tree. HM063908 and KC951571 are not isolated strains but also used in small tree construction (shown in Maroon). See Fig S6 for other information.

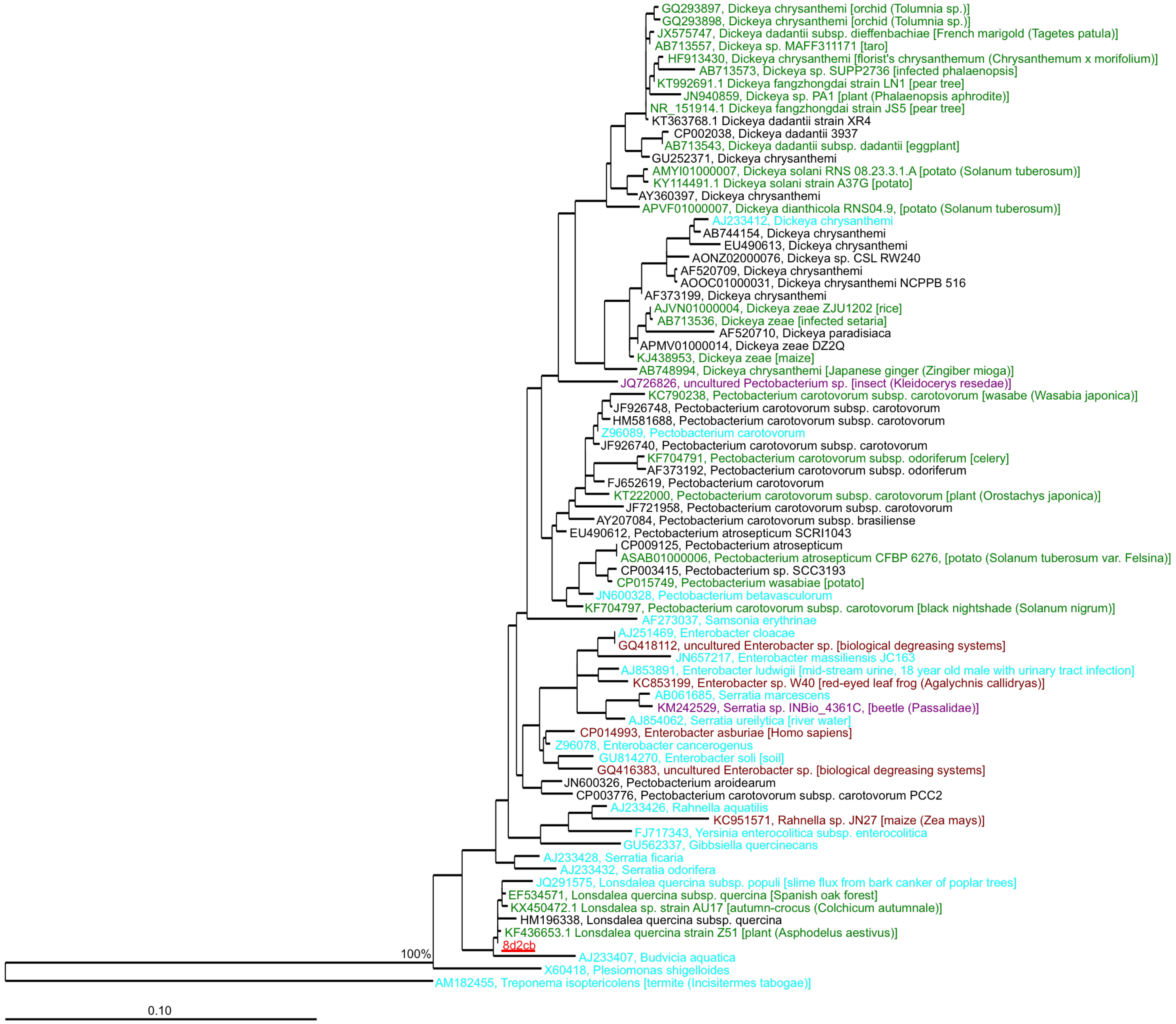

Fig S8. Phylogenetic tree containing 8d2cb and relative Enterobacteriaceae. Names in cyan are isolated strains, which were used in construction of small tree. CP014993, GQ416383, GQ418112, KC853199, and KC951571 are not isolated strains but also used in small tree construction (shown in Maroon). See Fig S6 for other information.

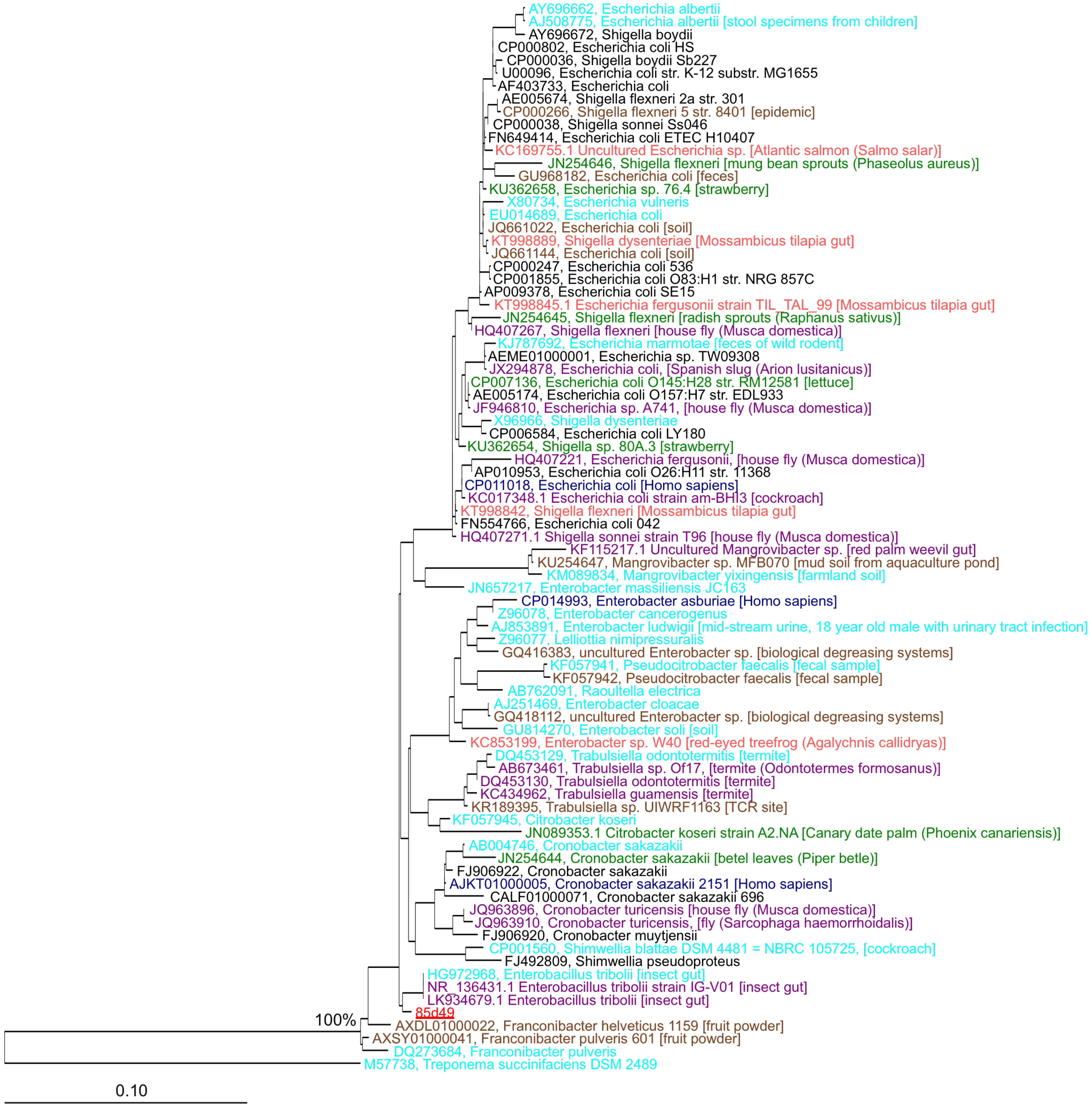

Fig S9. Phylogenetic tree containing 85d49 and relative Enterobacteriaceae. M57738 was used as an outgroup. See Fig S6 for other information.

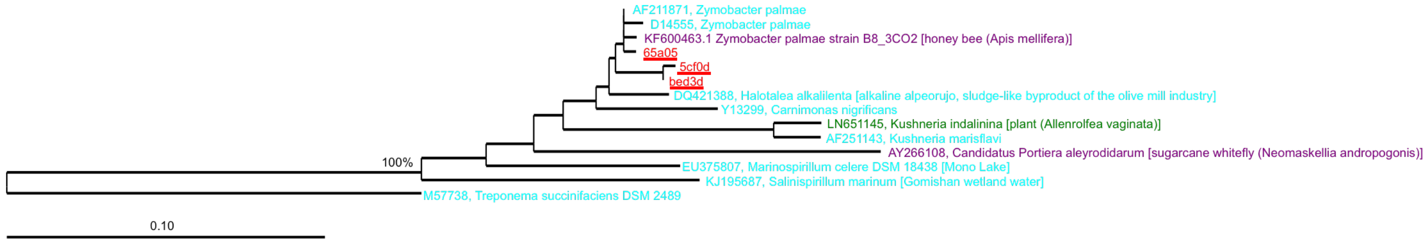

Fig S10. Phylogenetic tree containing 65a05, 5cf0d, bed3d and relative Halomonadaceae. See Fig S9 for other information.

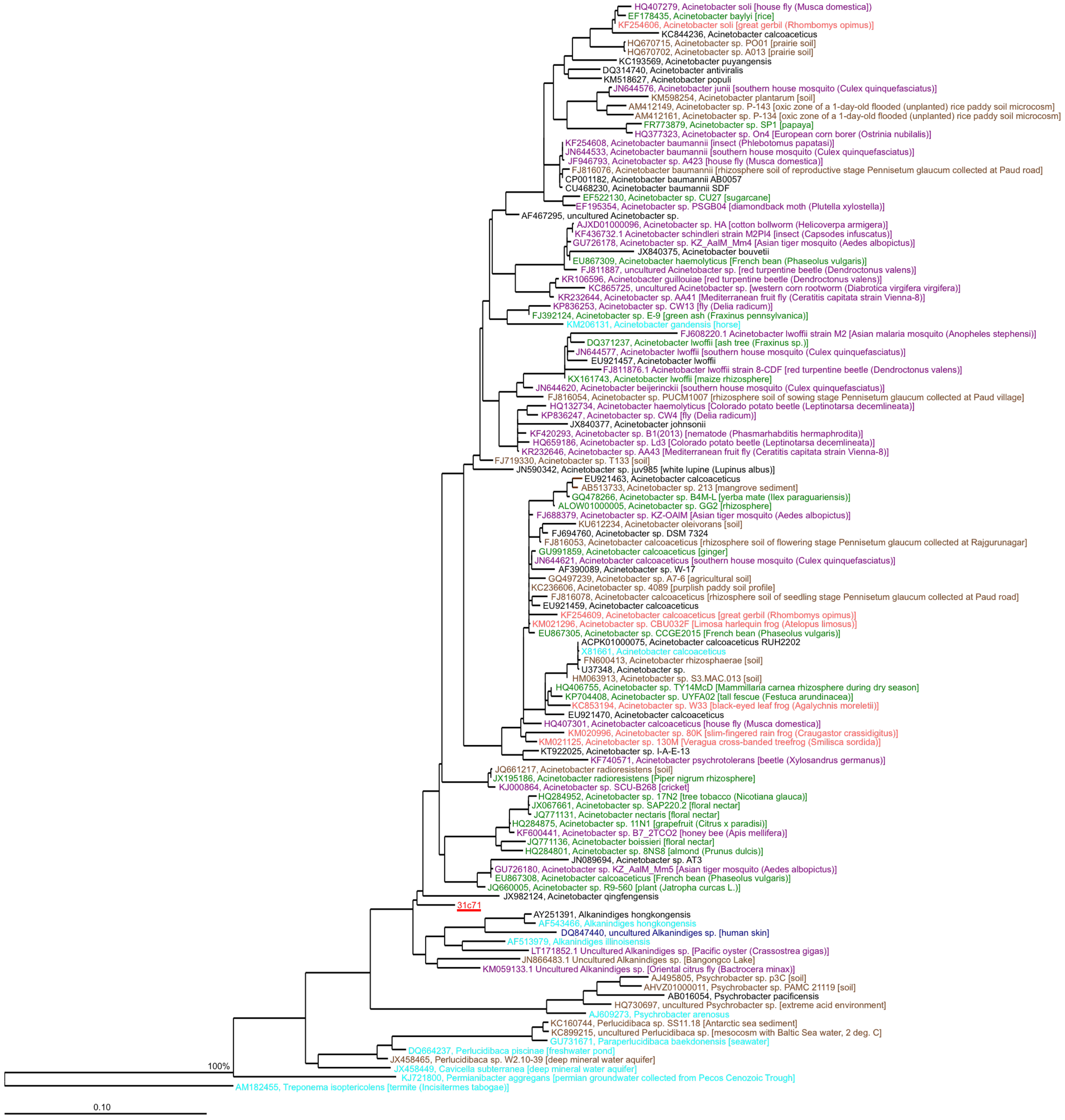

Fig S11. Phylogenetic tree containing 31c71 and relative Moraxellaceae.
See Fig S6 for other information.

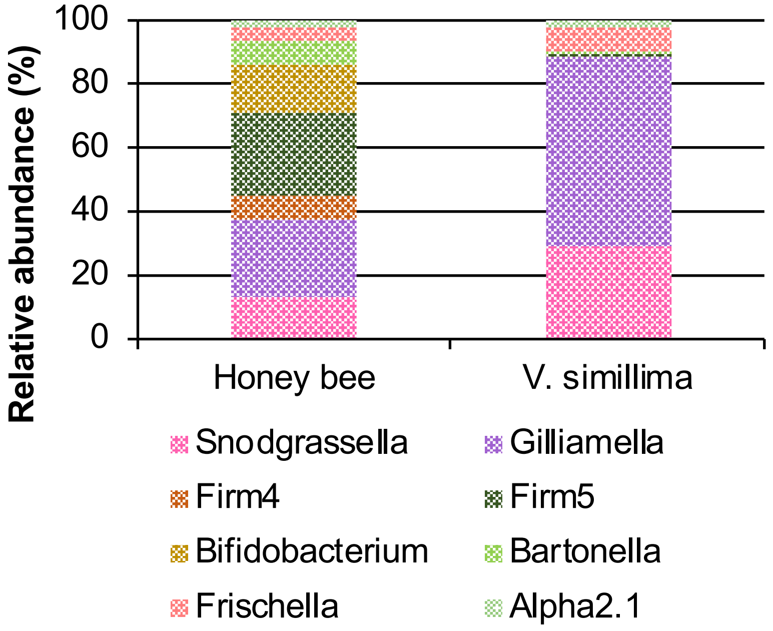

Fig S12. Comparison of honey bee gut microbiome and their relatives in the hornet gut. Relative abundance of major eight genera in the honey bee gut and their relatives in the *V. simillima* gut. Plots are scaled to 100%.

Table S1. Top hit sequences of hornet core OTUs in BLAST search

OTU ID	e-value	Identity (%)	Accession number	Organism	Isolation source	Host
80f1d	3.00E-131	100	NR_126291	Gibbsiella greigii	n. d.	n. d.
			NR_118387	Gibbsiella papilionis	intestinal tract	Mycalesis gotama
			GU826700	Serratia sp. TN49	n. d.	Batocera horsfieldi (Hope)
			NR_108121	Gibbsiella dentisursi	bear oral cavity	n. d.
			GU562342	Gibbsiella quercinecans	n. d.	n. d.
			NR_117526	Gibbsiella quercinecans	n. d.	n. d.
			GQ417538	uncultured Serratia sp.	biological degreasing systems	n. d.
			EF534572	Serratia sp. N-78b	Spanish oak forest	n. d.
85d49	7.00E-132	100	NR_126291	Gibbsiella greigii	No description	n. d.
			NR_118387	Gibbsiella papilionis	intestinal tract	Mycalesis gotama
			GU826700	Serratia sp. TN49	n. d.	Batocera horsfieldi (Hope)
			NR_108121	Gibbsiella dentisursi	bear oral cavity	n. d.
			GU562342	Gibbsiella quercinecans	n. d.	n. d.
			NR_117526	Gibbsiella quercinecans	n. d.	n. d.
			GQ417538	uncultured Serratia sp.	biological degreasing systems	n. d.
			EF534572	Serratia sp. N-78b	Spanish oak forest	n. d.
1d321	3.00E-131	100	AY350737	Zymomonas mobilis subsp. pomaceae	n. d.	n. d.
a1527	1.00E-128	99	JX228434	bacterium 37-L049660-122-017-E05	Anderson impactor sample from plate bac15	n. d.
	6.00E-127	99	CP028349	Pantoea vagans	blood of an infant	Homo sapiens
			MH379795	Pantoea sp.	plants of soil contaminated by high concentrations of toxic metals, lead	n. d.
			MG831371	uncultured Enterobacteriaceae bacterium	n. d.	n. d.
f26d5	3.00E-131	100	KX450472	Lonsdalea sp.	pollen	Colchicum autumnale
			JN600327	Pectobacterium aroidearum	n. d.	n. d.
			CP003776	Pectobacterium carotovorum subsp. carotovorum PCC21	n. d.	n. d.
			JQ046877	uncultured bacterium	phyllosphere	apple cultivar Gala
			JF311446	Lonsdalea britannica	n. d.	n. d.
			NR_118127	Lonsdalea britannica	n. d.	n. d.
			HM196339	Lonsdalea quercina	n. d.	n. d.
			HM150665	Pectobacterium carotovorum subsp. carotovorum	n. d.	n. d.
			HM179991	Pectobacterium carotovorum subsp. carotovorum	soil	n. d.
			GQ915088	Erwinia sp. AaMG18	midgut	Aedes albopictus
			EF534571	Lonsdalea quercina	Spanish oak forest	n. d.
			DQ785511	Pectobacterium carotovorum (Erwinia carotovora)	n. d.	n. d.
			DQ298132	Lonsdalea quercina	plant thorns	n. d.
			DQ068843	uncultured bacterium	GI tract sample, freshly caught antlion, OTU8	Myrmeleon mobilis
8d2cb	7.00E-132	100	KX450472	Lonsdalea sp.	pollen	Colchicum autumnale
			JN600327	Pectobacterium aroidearum	n. d.	n. d.
			CP003776	Pectobacterium carotovorum subsp. carotovorum PCC21	n. d.	n. d.
			JQ046877	uncultured bacterium	phyllosphere	apple cultivar Gala
			JF311446	Lonsdalea britannica	n. d.	n. d.
			NR_118127	Lonsdalea britannica	n. d.	n. d.
			HM196339	Lonsdalea quercina	n. d.	n. d.
			HM150665	Pectobacterium carotovorum subsp. carotovorum	n. d.	n. d.
			HM179991	Pectobacterium carotovorum subsp. carotovorum	soil	n. d.
			GQ915088	Erwinia sp. AaMG18	midgut	Aedes albopictus
			EF534571	Lonsdalea quercina	Spanish oak forest	n. d.
			DQ785511	Pectobacterium carotovorum (Erwinia carotovora)	n. d.	n. d.
			DQ298132	Lonsdalea quercina	plant thorns	n. d.
			DQ068843	uncultured bacterium	GI tract sample, freshly caught antlion, OTU8	Myrmeleon mobilis
65a05	3.00E-131	100	KF600463	Zymobacter palmae	bee bread food stores	Apis mellifera
			NR_041786	Zymobacter palmae	n. d.	n. d.
			HM110603	uncultured gamma proteobacterium	single abdomen	Chalybion californicum
			AF211871	Zymobacter palmae	n. d.	n. d.
5cf0d	3.00E-124	98	KF600463	Zymobacter palmae	bee bread food stores	Apis mellifera
			NR_041786	Zymobacter palmae	n. d.	n. d.
			HM110603	uncultured gamma proteobacterium	single abdomen	Chalybion californicum
			NR_043806	Halotalea alkalilenta	alkaline alpeorujo, sludge-like byproduct of the olive mill industry	n. d.
			AF211871	Zymobacter palmae	n. d.	n. d.
31c71	1.00E-129	99	KC137147	uncultured bacterium	adult worker ant	Polyrhachis monteithi voucher CSM0709
	1.00E-128	99	LC333521	Acinetobacter sp.	floral nectar	n. d.
bed3d	8.00E-125	98	KF600463	Zymobacter palmae	bee bread food stores	Apis mellifera
			NR_041786	Zymobacter palmae	n. d.	n. d.
			HM110603	uncultured gamma proteobacterium	single abdomen	Chalybion californicum
			NR_043806	Halotalea alkalilenta	alkaline alpeorujo, sludge-like byproduct of the olive mill industry	n. d.
			AF211871	Zymobacter palmae	n. d.	n. d.
5c53b	3.00E-131	100	NR_149810	Gilliamella bombicola	bumble bee gut	n. d.
			KM454417	Gilliamella apicola	n. d.	n. d.
			CP007445	Gilliamella apicola	n. d.	n. d.
			KF600321	Gilliamella apicola	alimentary tract hindgut	Apis mellifera
			JQ389991	uncultured gamma proteobacterium	bee gut	Bombus bohemicus
f9b86	7.00E-132	100	NR_149810	Gilliamella bombicola	bumble bee gut	n. d.
			KM454417	Gilliamella apicola	n. d.	n. d.
			CP007445	Gilliamella apicola	n. d.	n. d.
			KF600321	Gilliamella apicola	alimentary tract hindgut	Apis mellifera
			JQ389991	uncultured gamma proteobacterium	bee gut	Bombus bohemicus

Sequences with the highest similarity to core OTUs are shown. Information on isolation source and host are referred in GenBank. The OTU IDs were shortened to the last five characters. Core OTUs unique to *V. mandarinia*, *V. simillima*, and shared between both are highlighted in blue, yellow, and green, respectively.
